# AutoGIS-driven solar pond site selection for water treatment in Africa aligned with the NEXUS framework

**DOI:** 10.1038/s41598-025-01778-6

**Published:** 2025-05-22

**Authors:** Mahmoud Fatehy Altahan, Mohamed Nower

**Affiliations:** 1 Water Management Research Institute (WMRI), National Water Research Center (NWRC), El-Qanater El-Khairia, 13621 Egypt; 2 Central Laboratory for Environmental Quality Monitoring (CLEQM), National Water Research Center (NWRC), El-Qanater El-Khairia, 13621 Egypt

**Keywords:** AutoGIS processing, DBSCAN clustering, Solar ponds, Site selection, Python programming

## Abstract

Access to clean and safe water remains a critical challenge across many regions in Africa. This study investigates the potential of solar ponds as wastewater treatment facilities by employing AutoGIS processing and DBSCAN clustering to identify suitable development sites across the African continent and its five regions: North, East, West, Central, and Southern Africa. By integrating environmental data such as solar radiation, wind speed, temperature, clear sky, cloud cover, and precipitation, this research highlights the effectiveness of geospatial tools in addressing clean water access issues. The findings reveal distinct suitability patterns for solar pond development at both continental and regional levels. Key environmental factors, notably direct normal radiation (DNR), temperature, and wind speed, consistently expanded the areas identified as suitable, while cloud cover demonstrated a positive effect. Precipitation showed minimal variation, particularly in Central Africa. Importantly, the study underscores the capability of DBSCAN clustering in handling large datasets, filtering noise, and capturing nuanced regional differences, which varied significantly from continent-wide trends. By streamlining the site selection process, this research offers practical insights into leveraging geospatial technologies to address water access challenges in Africa. The integration of AutoGIS and DBSCAN provides a scalable approach for analyzing complex environmental datasets, paving the way for more informed and sustainable development of wastewater treatment solutions across diverse regions.

**Supplementary Information:**

The online version contains supplementary material available at 10.1038/s41598-025-01778-6.

## Introduction

The global reliance on non-renewable energy sources has led to significant environmental impacts, with the combustion of fossil fuels contributing to 90% of worldwide greenhouse gas emissions. In response, there has been a growing emphasis on the development of powerful and sustainable energy alternatives, particularly renewable energy sources like solar powers^1,2^. The utilization of solar ponds for wastewater treatment in Africa holds immense potential, as it intersects with the critical water, energy, and food sectors, as outlined in the NEXSUS frameworks^3–5^. Addressing the increasing demand for these essential resources, while mitigating the intensifying effects of climate change present a significant challenge^6^. The integration of solar ponds for wastewater treatment in Africa not only addresses the pressing need for clean water access but also aligns with the overarching goals of the NEXUS frameworks^4,7^, which aim to integrate these sectors in a cost-effective, efficient, and sustainable manner^8,9^.

Solar ponds, large saltwater lakes used to collect and store solar energy, have emerged as crucial renewable energy technology^10^. Due to their simplicity, cost-effectiveness, and emission-free nature, these systems are attractive options for electricity production, particularly in Africa.. Beyond electricity, they are also used for wastewater treatment, aiding water purification and reducing pollution, which is vital in regions facing freshwater shortages, benefiting agriculture and the food supply chain. Site selection for solar pond development leverages remote sensing data to optimize land suitability, with Python playing a key role in this process. However, studies on solar pond site selection remain scarce. Solar ponds, reliant on solar radiation, are crucial in renewable energy as they provide a sustainable and clean energy source^11^. Beyond electricity, they are also used for wastewater treatment, aiding water purification and reducing pollution^12^, which is vital in regions facing freshwater shortages, benefiting agriculture and the food supply chain^13^. Site selection is crucial for identifying suitable lands for solar pond development and integrating remote sensing environmental data. In this regard, Python software plays a pivotal role in the strategy for evaluating potential sites^14^, facilitating the realization of NESUS strategies^15^. Despite the importance of this topic, literature on site selection for solar ponds at local or regional scales remains scarce.

Solar ponds have been researched in various African countries, primarily to assess their efficiency for diverse applications. For instance, in Angola, Cardoso et al. conducted a thermal analysis of a 1-hectare solar pond, revealing significant energy production after two years of operation^16^. Similarly, Rghif et al. explored heat and mass transfer in a solar pond in Morocco, demonstrating that adjustments to the Defour coefficient enhanced performance^17^. In Egypt, Madkour et al. investigated the use of solar ponds for recovering brine solutions from reverse osmosis processes^18^.

The selection of sites for solar pond development relies heavily on environmental remote sensing data, such as direct normal radiation and wind speed.

These factors ensure optimal solar exposure and thermal conditions, maximizing energy capture and sustainability. While numerous studies have addressed site selection for renewable energy plants, many methods, including the spherical fuzzy AHP^19^, introduce complexity and subjectivity that can hinder effective decision-making^20^.

This study presents a novel methodology utilizing Python programming to develop an AutoGIS algorithm for site selection across Africa. This approach incorporates over 18,000 data points, marking the first comprehensive analysis for the continent, as previous research focused on individual countries. By employing density-based clustering of applications with noise (DBSCAN), we aim to identify optimal locations for solar pond development, addressing both wastewater treatment and renewable energy generation. This research aligns with the NEXUS framework, emphasizing the need to balance water and energy resources to meet Africa’s growing demands sustainably.

## Methods

**Fig. 1:**
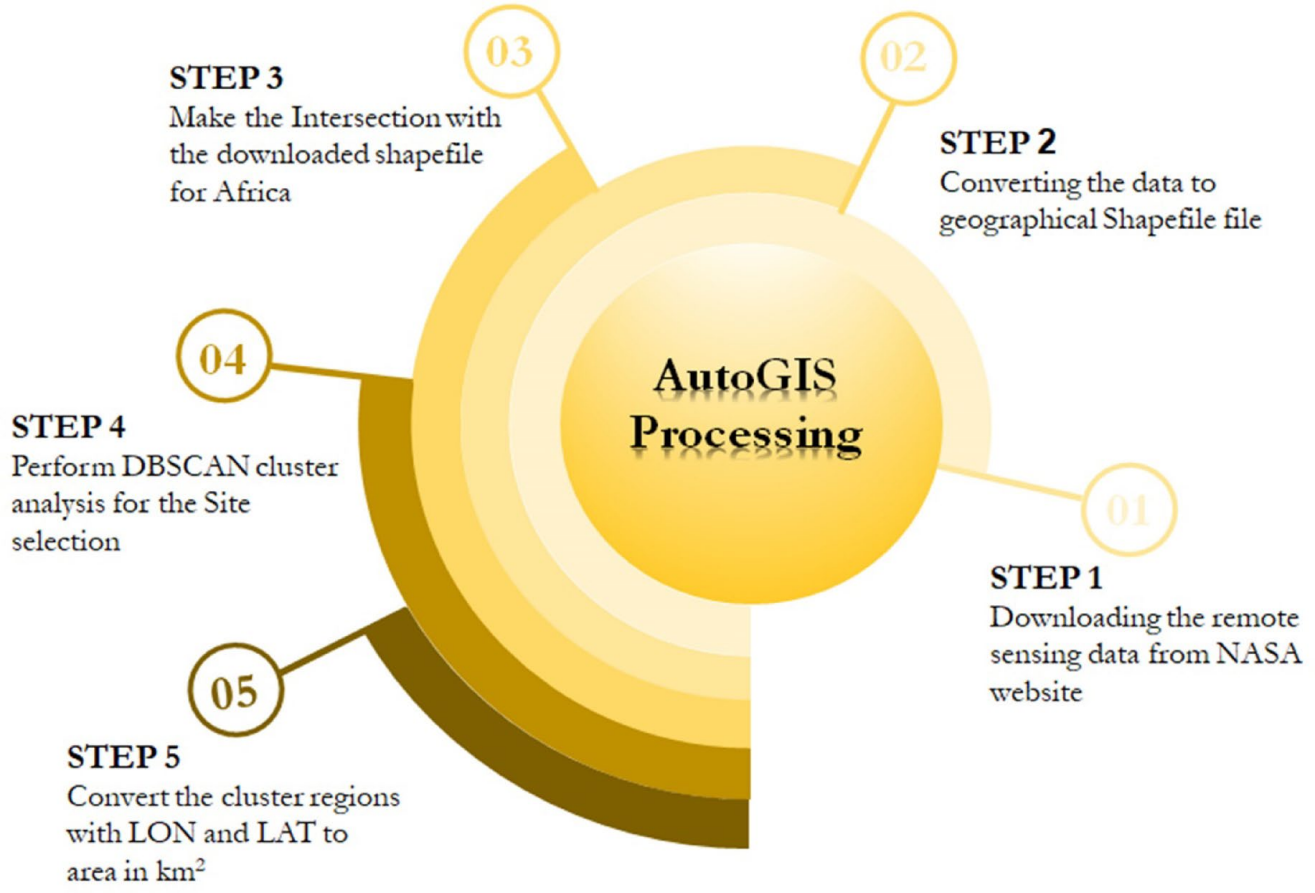
Infographic diagram illustrating the 5-step process for site selection for solar pond development in Africa via Python software implemented in DBSCAN.

The geospatial dataset processing proceeded according to the flowchart illustrated in Fig. 1, encompassing a five-step procedure. Initially, the data were sourced from the NASA Prediction of Worldwide Energy Resources (NASA PPOWER) website. Next, the data were transformed into shapefiles, aligning with the second step in the flowchart. Following this transformation, the shapefile was intersected with a downloaded shapefile that delineates the boundaries of Africa, which is represented in the third step of the flowchart.

Subsequently, a DBSCAN clustering analysis was conducted for site selection, effectively organizing land suitability into clusters, as indicated in the fourth step. This clustering process is crucial for understanding the relationships between different land areas. Finally, the areas for the coordinates within the selected suitable and highly suitable regions were computed, completing the fifth step of the flowchart. The data were then spatially reviewed, followed by cluster analysis using DBSCAN, which identified five main clusters that categorize the optimal locations for solar pond development. Ultimately, the coordinates for the areas deemed highly suitable for site selection were calculated in the area calculation section.

### Data acquisition

The remote sensing data were downloaded from the Power Project on the NASA website covering a period from 2000 to 2020. The spatial environmental data were obtained from the NASA website with a spatial resolution of 0.5 degrees. The dataset included solar radiation (direct normal radiation) in kW-hr/m^2^/day, temperature at 2 m in °C, wind speed at 10 m, cloud amount in percentage, clear-sky insolation clearness index (dimensionless), and precipitation in mm/day. Solar radiation, particularly direct normal radiation (DNR), is crucial for solar pond development, as it measures the solar energy reaching the Earth when the sun is directly overhead^21^. Temperature is also vital, as it influences the efficiency and performance of solar ponds^22^. Wind speed is assessed to evaluate its potential impact on the stability of the infrastructure^23^. Additionally, cloud cover and the clear-sky insolation clearness index (dimensionless) affect the solar radiation that reaches the surface^24^, while precipitation provides insights into water balance and solar pond maintenance^25^. The entire dataset for Africa was transformed into a list of geometric points using the longitude and latitude columns, resulting in the creation of a GeoDataFrame with a specified coordinate reference system (EPSG:32,651). This data was then converted into an ESRI shapefile, facilitating effective storage and manipulation of geographic information. Shapefiles for all of Africa and each country were obtained from the GDAM maps and data website.

**Fig. 2:**
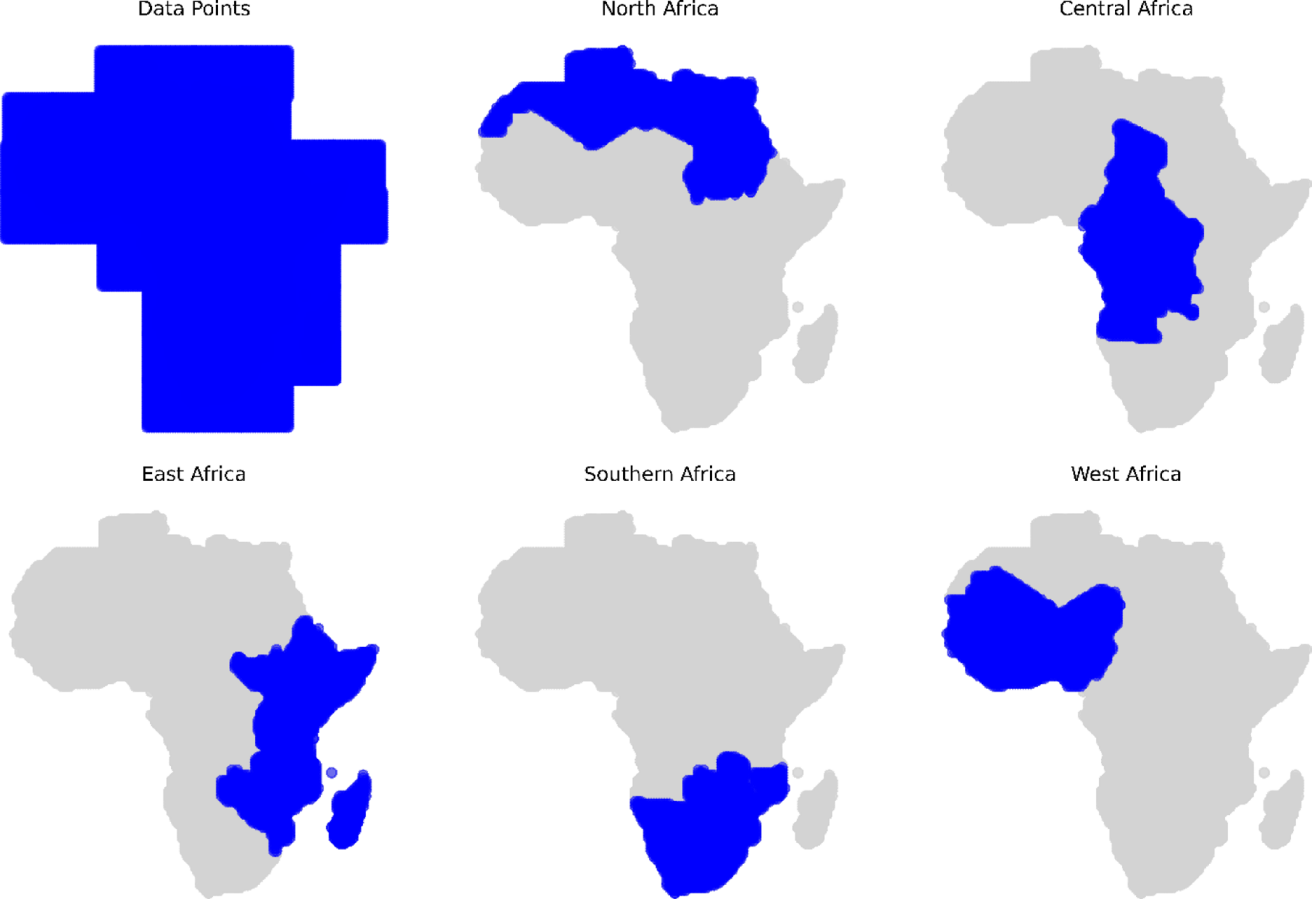
Overviews of the distribution of the downloaded datapoint from the NASA POWER website and five discrete regions of Africa (North Africa, Central Africa, East Africa, Southern Africa, and West Africa) in our gathered data.

To facilitate regional analysis and better capture geographic variability, countries were grouped into five distinct regions: North, East, West, Southern, and Central Africa. This regional discretization allows for targeted cluster analysis that accounts for the environmental and climatic differences across the continent. Shapefiles corresponding to each region were created by aggregating national shapefiles, and an intersection was performed between the environmental dataset and each regional boundary (as shown in Fig. 2). This process ensured that clustering was performed within spatially and environmentally coherent zones, improving the interpretability and relevance of the analysis.

### DBSCAN clustering

In the process of selecting a site, the density-based spatial clustering of application with noise (DBSCAN) algorithm plays a crucial role^26^. DBSCAN stands out from traditional clustering algorithms due to its flexibility in determining the number of clusters and its ability to identify outliers as noise rather than including them in a cluster^27^. Two key parameters, epsilon (Ɛ) and the minimum points (MinPts), are fundamental to the application of DBSCAN. Epsilon represents the radius for searching for neighboring points, while MinPts determines the minimum points within the epsilon neighborhood of the core point as part of the cluster^28^. Core points are defined as those with at least MinPts within their epsilon neighborhood, representing regions of high density^29^. Before the site selection process, minimum points were set MinPts = D + 1 where D is the number of features for each input combination. Where Ɛ value was indicates by plotting the distances to each point’s MinPts nearest neighbor, sorted in ascending order (k-distance plot). The “knee” or elbow in the plot, where the curve sharply increases, indicates the optimal ε value. This is the point where noise starts, and clusters end. While epsilon value was then also altered to ensure obtain only 4 clusters. Choosing to limit the number of clusters to 4 allows for a smaller epsilon value, which helps minimize the inclusion of noise points as separate clusters^30^. Table S1 show the epsilon values obtained from k-distance plot and the adapted Ɛ for Africa and five reginal regions. This results in a cleaner clustering output that emphasizes the primary structures of the data. Additionally, restricting the number of clusters improves computational efficiency for subsequent analyses and visualizations. The sites were categorized based on their suitability, with the most suitable sites characterized by high DNR, moderate temperatures, and minimal impact from other environmental factors.

### The area calculations.

Following the completion of the clustering analysis, the results were documented in an Excel spreadsheet, detailing the geospatial computations conducted using geographic coordinate data. The Python script utilized a collection of libraries, including pandas, geopy, shapely, and pyproj^31^. The area calculation process unfolded in several steps. It begins by reading the coordinates from specified sheets in an Excel file, converting these coordinates from latitude and longitude to a coordinated reference system specifically a Universal Transverse Mercator (UTM) (EPSG: 4326) projection according to Eq. 1.

1 $$X,Y=transform (Lattiude, Longitude)$$

The script then creates a polygon from the transformed points and computes its area in square meters according to Shoelace formula as shown in Eq. 2, converting this value to hectares and the results were stored in a new Excel sheet^32^.

2 $${A}_{{m}^{2}/10000}= \frac{1}{2} \left|\sum_{i=1}^{n}({X}_{i}{Y}_{i+1}-{X}_{i+1}{Y}_{i})\right|$$

The created Excel sheet showed the data in a well-organized and easily accessible manner.

## Results and discussion

**Fig. 3:**
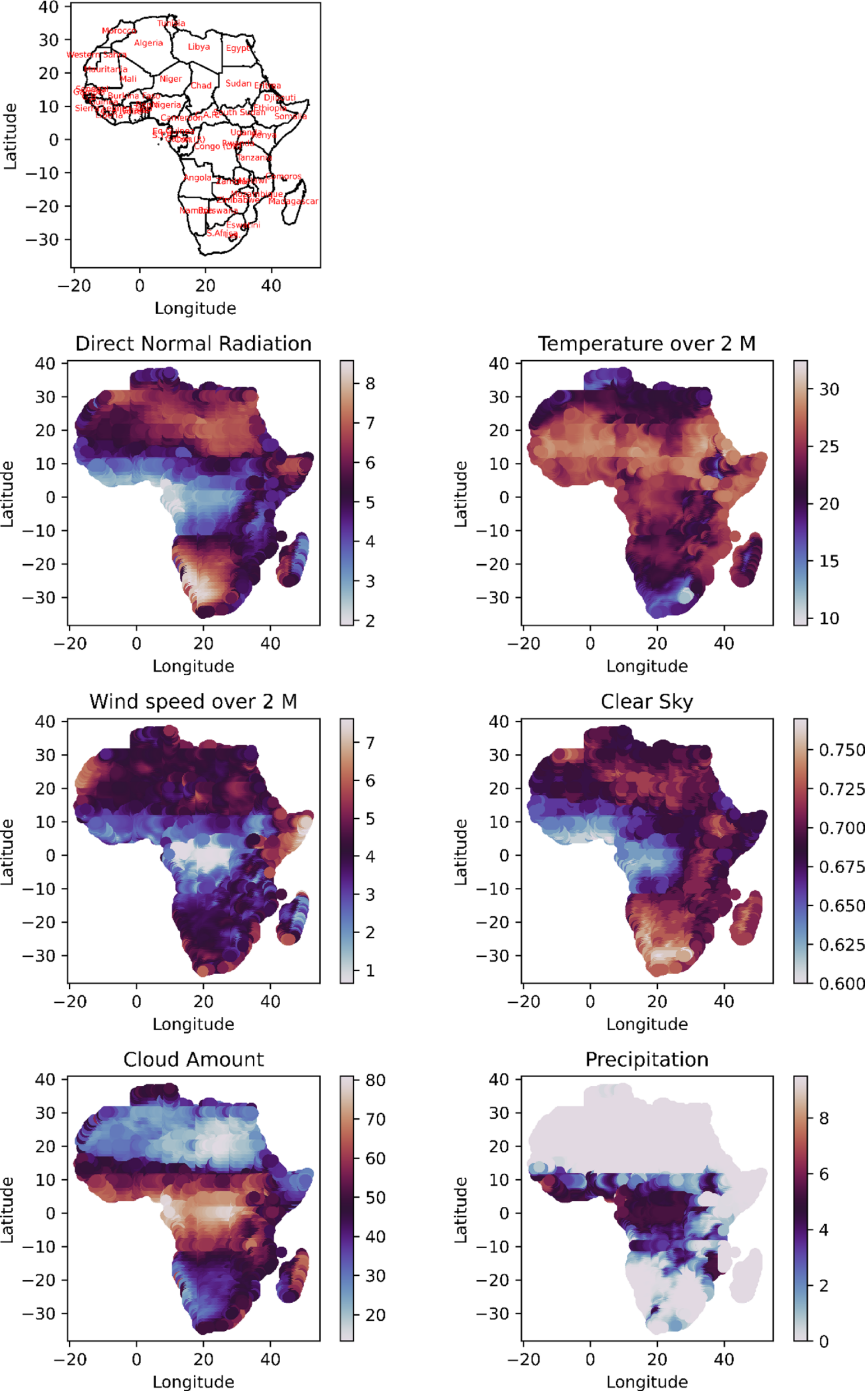
Visual representations showcasing the distribution patterns of key environmental parameters across the African continent, such as direct normal radiation (DNR), temperature at a height of 2 m, wind speed at a height of 10 m, clear sky conditions, cloud cover, and precipitation. The presented data represents annual averages.

The process of site selection for solar pond development is crucial for ensuring optimal performance and functionality. This approach involves identifying locations with high solar radiation, suitable temperature conditions, minimal environmental impact, and clear sky radiation. Other factors, such as temperature, wind speed, precipitation, and proximity to water resources, are also essential considerations. Direct normal radiation (DNR), which indirectly measures the portion of solar radiation reaching the Earth’s surface, is influenced by factors such as water vapor, aerosols, and clouds. These considerations are integral to the successful establishment and operation of solar ponds. The spatial distribution of the environmental remote sensing data across the study area, as depicted in Fig. 3, post intersection with the boundaries of Africa reveals notable patterns. In the northern region of Africa, particularly around the latitude of 10°N, solar radiation range from 5 to 7 Kw-hr/m^2^/day, mirroring a similar trend in the southern region at 15°S. The temperature distribution across 2 m has been relatively consistent at approximately 20 °C throughout Africa, except above 30°N and below 25°S, where the temperature ranges from 15 °C to 20 °C. The wind speed distribution over 2 m in m s^−1^ indicates peak values exceeding 5 m s^−1^ in the Horn of Africa, with values between 4 and 5 ms^-1^ observed around a latitude of 15°N. In the region spanning from a latitude of 10°N to 25°S, the wind speed values fall below 3 ms^-1^, reaching approximately 1 ms^-1^ around the equator. The spatial distribution of environmental data across Africa reveals favourable conditions for solar energy in the northern and southern latitudes (10°N to 15°S), wind energy in the Horn of Africa and regions around 15°N, and agricultural activities in moderate temperature zones (around 20°C), providing key insights for optimal site selection in renewable energy and agriculture^33^.

The dimensionless clear sky parameter exhibits a spatial distribution with a consistent range of more than 0.7 within the region defined by latitudes between 15°N and 15°S and longitudes from 25°E to 20°W. Cloud cover, expressed as a percentage, exhibited values less than 30% in North Africa at approximately 15°N and approximately 40% in southern Africa below 15°S. In the region spanning from 10°N to 15°S, the cloud cover exceeds 60%. The spatial distribution of precipitation for Africa indicates zero values, excluding the region between 10°N and 15°S, where values range from 2 to 5 mm/day^-1^. This also reflected the increase in the epsilon value from 0.06 to 0.9, where the epsilon value indicates the radius of the neighbouring points, indicating the coherence of the points used to create the clusters.

**Fig. 4:**
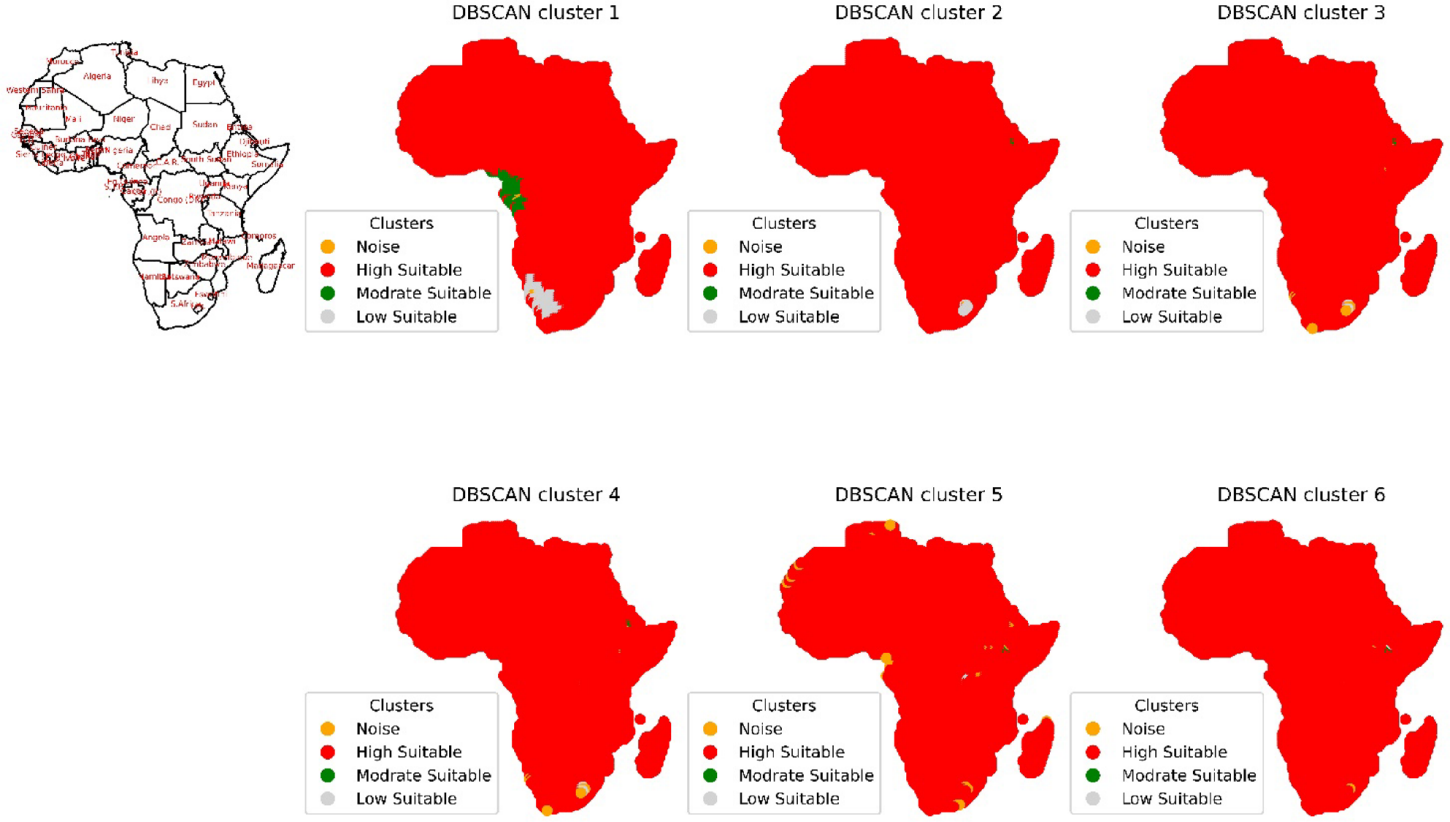
Illustrations displaying the distribution patterns resulting from the application of DBSCAN cluster analysis to various combinations of environmental parameters in Africa. The depicted outputs represent the annual means of the analysis.

**Fig. 5:**
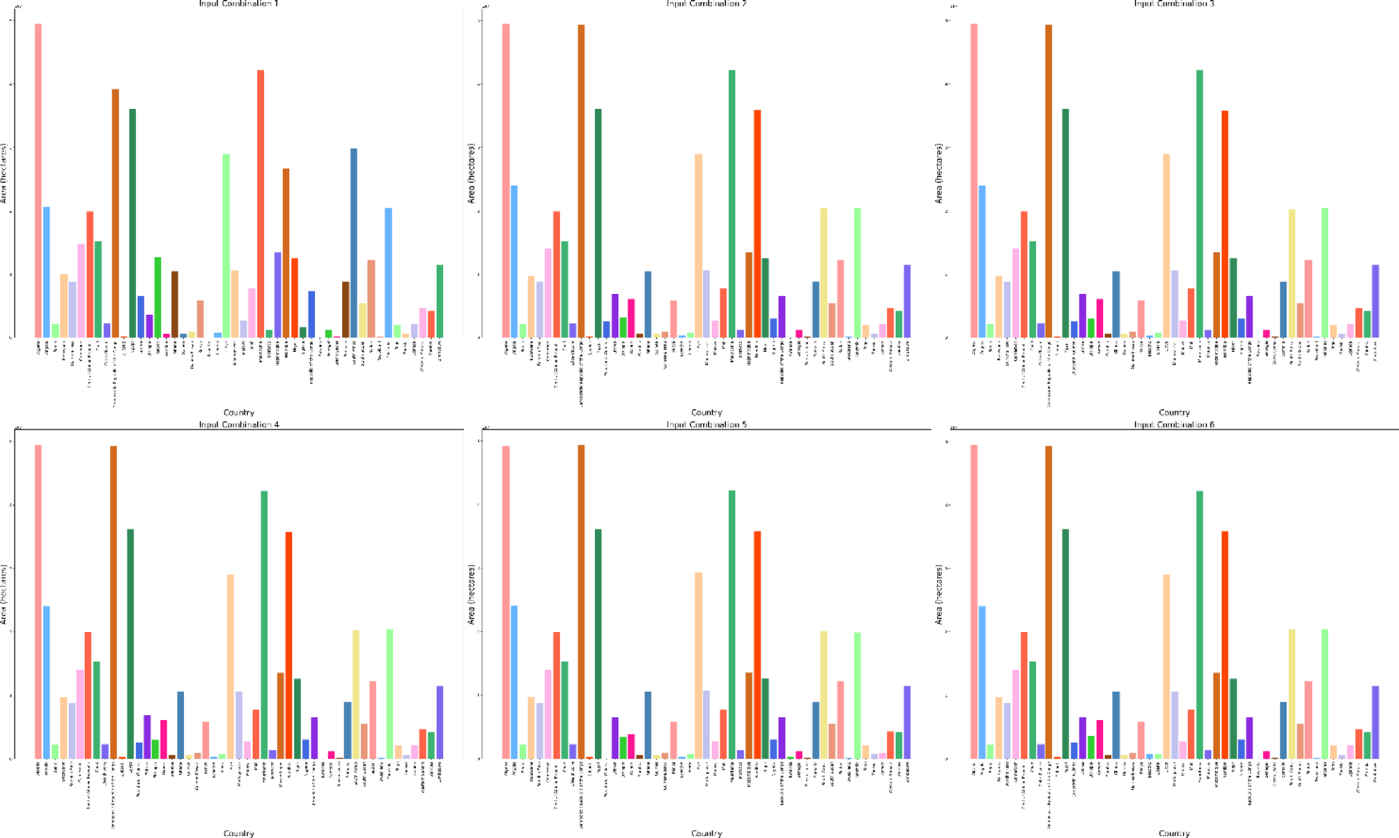
Area of the highly suitable cluster for solar pond development site selection for each country across Africa, measured in hectares for six input combinations.

**Fig. 6:**
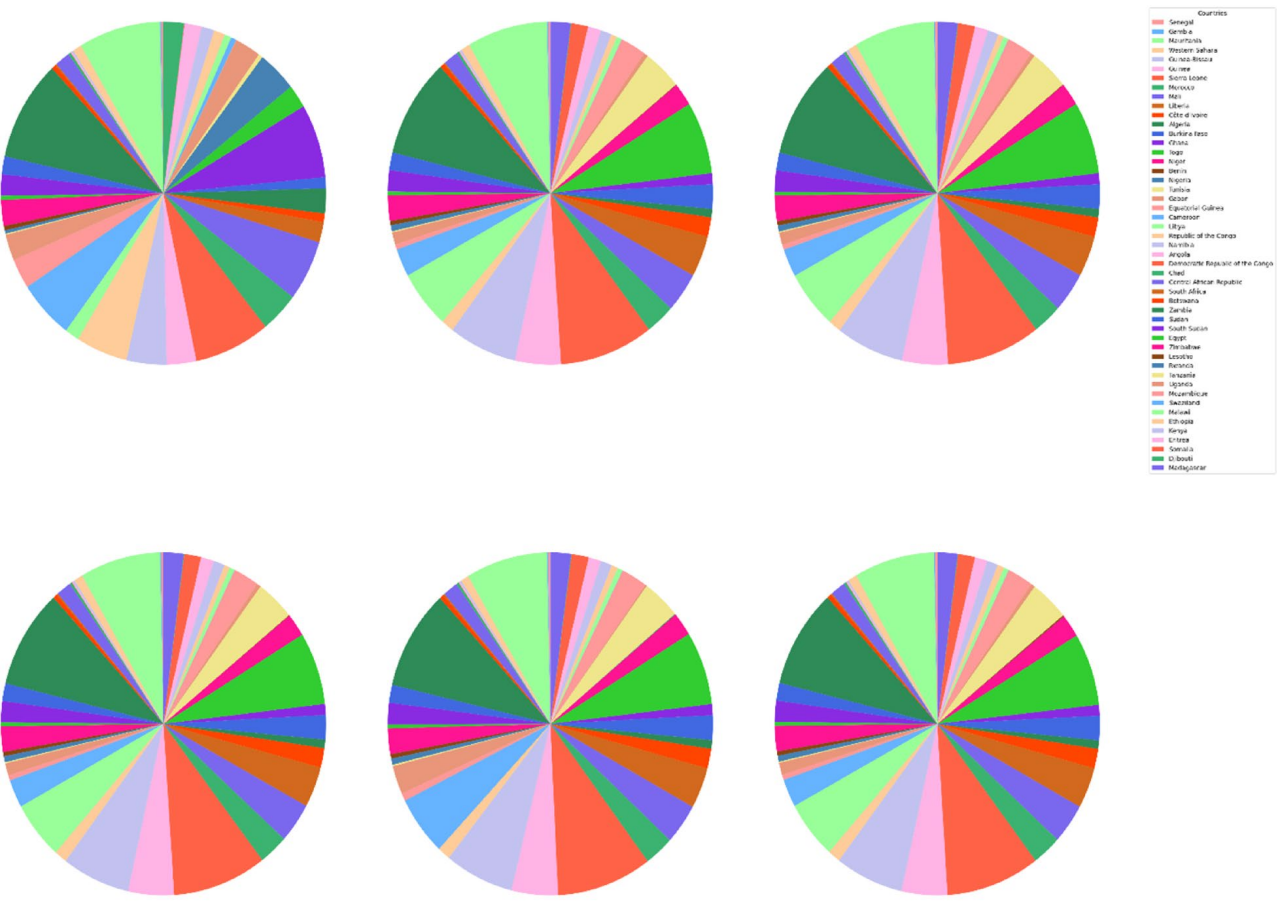
Pie charts for the percentages of the area occupation for the countries of the whole of Africa for the high suitability cluster for solar ponds development site selection for six input combinations from left to right and from top to bottom.

The DBSCAN clustering algorithm was applied to different input combinations of environmental remote sensing data (6 input combinations), as shown in Fig. 4. The first input combination started with the DNR, which has a high impact on the functionality of the solar pond. The DNR distribution data will help to determine the sunlight available for solar pond operation and energy storage. Then, for a cluster with an input combination, the temperature is considered in terms of the DNR and temperature. Temperatures have a greater impact DNR on the performance of solar ponds than does DNR. Temperature data are highly essential because they help in understanding the thermal characteristics of the site. Moreover, this approach is essential for evaluating ambient temperature conditions. The temperature data will help directly impact the heat exchange processes within solar ponds. Subsequently, the wind speed was included in the input combination for DBSCAN clustering for the site selection process. The wind speed data could be valuable for assessing local wind speed conditions. The wind speed data could help to understand the effect of convective heat loss from the solar pond surface. For Input combination 4, the clear Sky parameter was included in the previous inputs in the previous combination. The cloud amount parameter was included in input combination 5 for the application of the DBSCAN clustering algorithm. Clear sky radiation data are highly essential and are assessed based on various atmospheric parameters, including clear sky measurements and cloud amount measurements. The cloud amount usually negatively impacts the amount of solar radiation that reaches the Earth’s surface. For the sixth input combination, the precipitation parameter was included to ensure the water supply and management of the solar ponds. The results for input combination 1 (DNR only) revealed the identification of four clusters, labeled "Noise," “High Suitable”, "Moderate Suitable," and "Low Suitable." This analysis evaluates the suitability of various site locations for solar pond development using DBSCAN clustering, with an epsilon value set at 0.06. The process resulted in the creation of multiple polygons covering a total area of 28.9 × 10^8^ hectares across the entirety of Africa. The primary regions identified include Algeria, the Democratic Republic of Congo, Mauritania, and Egypt, as illustrated in Figs. 5 and 6.

In Namibia and South Africa, the multipolygon generated by DBSCAN clustering labeled as “Low Suitable” extends from 15.25°E, 22.25°S to 19.75°E, 22.25°S and from 19.75°E, 28.25°S to 20.75°E, 32.75°S with an area of 5.2 × 10^7^ hectares.

In the following input combination, four clusters like the previous ones were identified through the application of DBSCAN clustering with an epsilon value of 0.9. The cluster designated as “High Suitable” was found across Africa, encompassing an area of 2.99 × 10^8^ hectares. The main countries represented in this cluster are Algeria, the Democratic Republic of Congo, Mauritania, Namibia, and Egypt, as depicted in Figs. 5 and 6. The cluster labeled “Moderate Suitable” forms a multipolygon area with coordinates of 40.75°E, 12.75°N to 41.25°E, and 13.75°N in Ethiopia, covering an area of 1.76 × 10^5^ hectares. Additionally, another cluster labeled “Low Suitable” covers an area of 7.5 × 10^5^ hectares in Lesotho, with coordinates ranging from 28.25°E, 29.25°S to 29.25°E, 30.25°S. These findings highlight the effectiveness of the DBSCAN clustering algorithm in identifying suitable locations for solar pond development based on the inclusion of precipitation data in the input combination.

For the third input combination, almost the same results were obtained as for the previous input combination. The application of DBSCAN clustering with an epsilon value of 0.9 yielded 4 clusters, the “High Suitable” cluster obtained as before over whole Africa with an area of 2.99 × 10^8^ hectares which main countries represented in Algeria, the Democratic Republic of Congo, Mauritania, Namibia, and Egypt. Where the cluster labeled “Modrate Suitable” had coordinates of 41.25°E, 12.75°N to 40.75°E, and 13.75°N in Ethiopia, with a multipolygon area of 1.76 × 10^5^ hectares. Another cluster, labeled “Low Suitable”, had multiple polygons with coordinates of 28.75°E, 28.75°S to 28.75°E, 29.75°S and an area of 5.6 × 10^5^ hectares. Similar results were obtained for the fourth input combination in Ethiopia and Lesotho, with the application of DBSCAN clustering using an epsilon value of 0.9.

For the fifth input combination, 4 clusters were observed, with the application of DBSCAN clustering using an epsilon value of 1.4. A cluster labeled as “High Suitable” created an area of 29.9 × 10^8^ hectares spread primarily in Algeria, Democratic Republic of Congo, Mauritania, Namibia and Egypt. The “Moderate Suitable” cluster had multiple polygons with coordinates of 38.75°E, 6.25°N to 39.75°E, 6.75°N and 35.75°E and 0.25°S to 35.75°E, 0.75°S, covering an area of 2.5 × 10^6^ hectares in Ethiopia and Kenya. Another cluster, labeled “Low Suitable”, had coordinates of 29.25°E, 1.25°S to 28.75°E, 2.75°S and 29.25°E, 1.75°S to 29.75°E, and 1.75°S in the Democratic Republic of the Congo and Rwanda, with an area of 8.5 × 10^5^ hectares.

In the sixth input combination, 4 clusters were derived using DBSCAN with an epsilon value of 2. The cluster labeled as “High Suitable” with an area similar as before with a value of 29.9 × 10^8^ hectares with the same primary countries. A cluster labeled “Moderate Suitable” had coordinates ranging from 38.75°E, 6.25°N to 39.75°E, 6.75°N in Ethiopia and Kenya, with a multipolygon area of 2.5 × 10^6^ hectares. However, no specific area was identified for the cluster termed "Low Suitable."

**Fig. 7:**
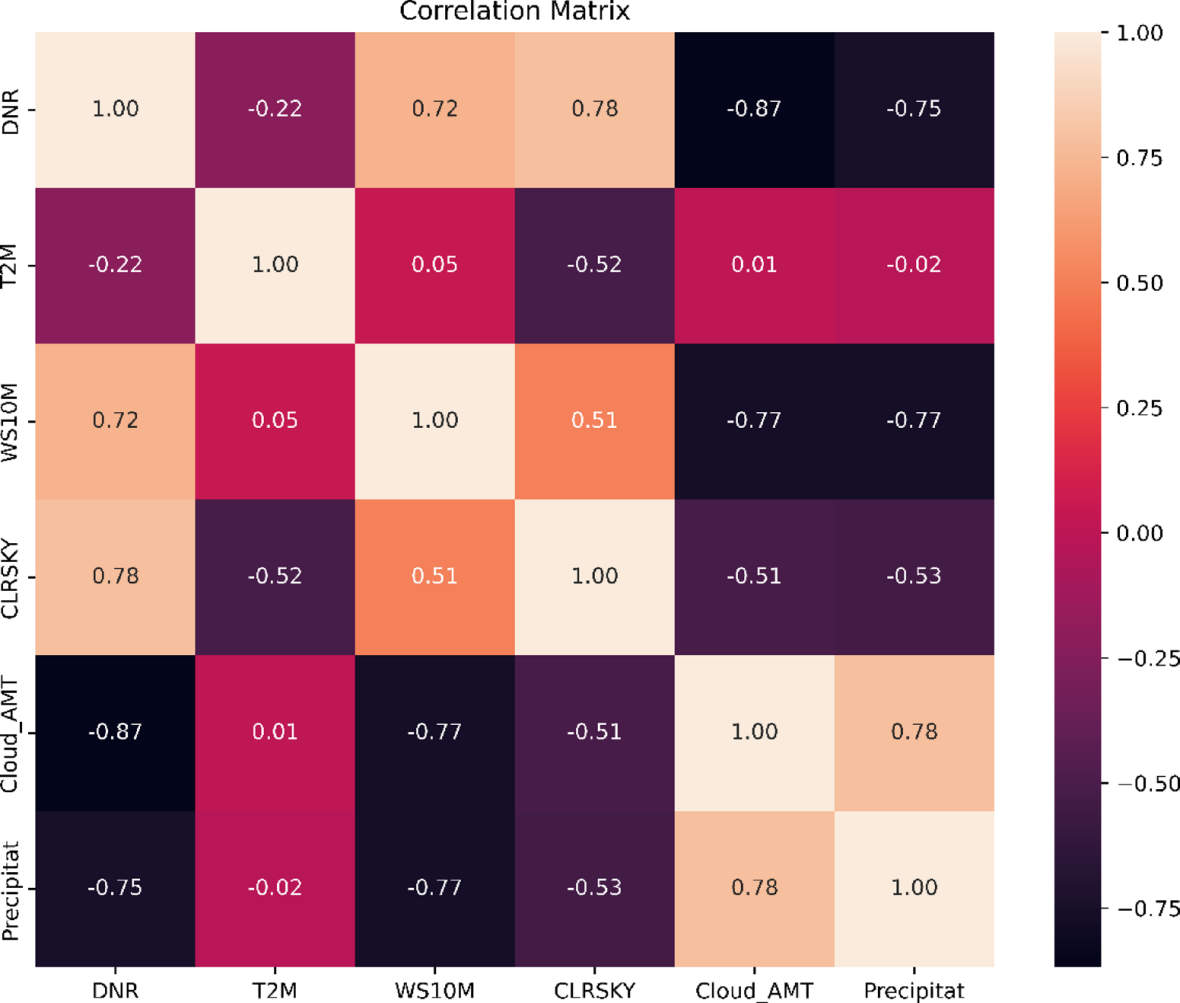
Correlation matrix for the environmental remote sensing data for all of Africa.

Where the epsilon values were reported as shown in Figure S1 and altered as indicated in Table S1. The findings indicate an increase in area when considering the temperature parameters in the clustering analysis, likely attributed to the decreased correlation between DNR and temperature, as indicated by a correlation coefficient of − 0.22 as shown in Fig. 7. The week correlation reflects that as temperature increases, DNR does not necessarily increase in a proportional manner. This results to a broader range of data points to be grouped into the cluster.

Furthermore, the influence of the wind speed and clear-sky conditions, with an epsilon value of 0.9 for the wind speed and a value of 0.9 for the clear-sky conditions. The correlation coefficients between the clear-sky data and DNR (0.78) and between the temperature data and clear-sky conditions (0.52) further contributed to these differences, as shown in Fig. 7. Additionally, there is a higher correlation coefficient between the cloud cover and DNR (with a negative value of 0.87) due to the inclusion of cloud cover data.

DBSCAN clustering analysis proves to be a valuable tool for clustering the site selection of solar pond development across Africa. However, its effectiveness is somewhat limited due to the wide variability of the data, which can impact the clarity of integrating the information into the clustering analysis. Therefore, a more practical approach would be to apply this cluster analysis to less dense data points within smaller-scale areas, specifically in five distinct regions: North Africa, East Africa, West Africa, Central Africa, and Southern Africa. This targeted approach allows for a more focused and precise analysis of site selection processes.

**Fig. 8:**
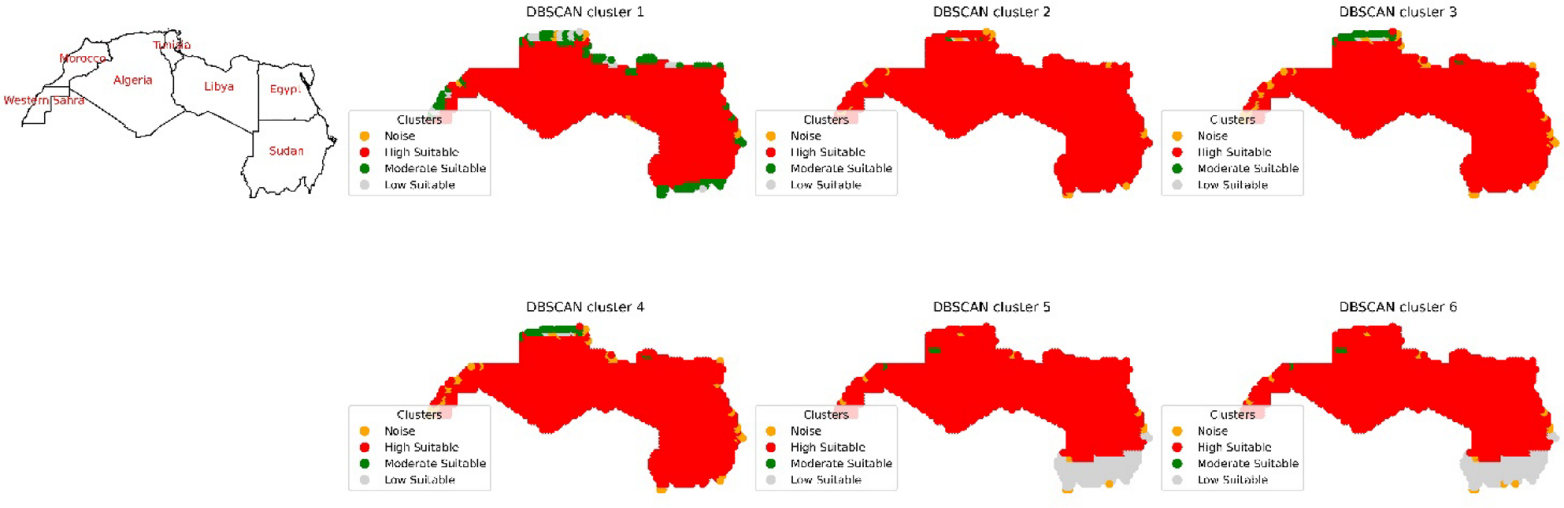
Overviews of the distributions of the outputs from the DBSCAN clusters under different combinations of environmental conditions in the North Africa region.

**Fig. 9:**
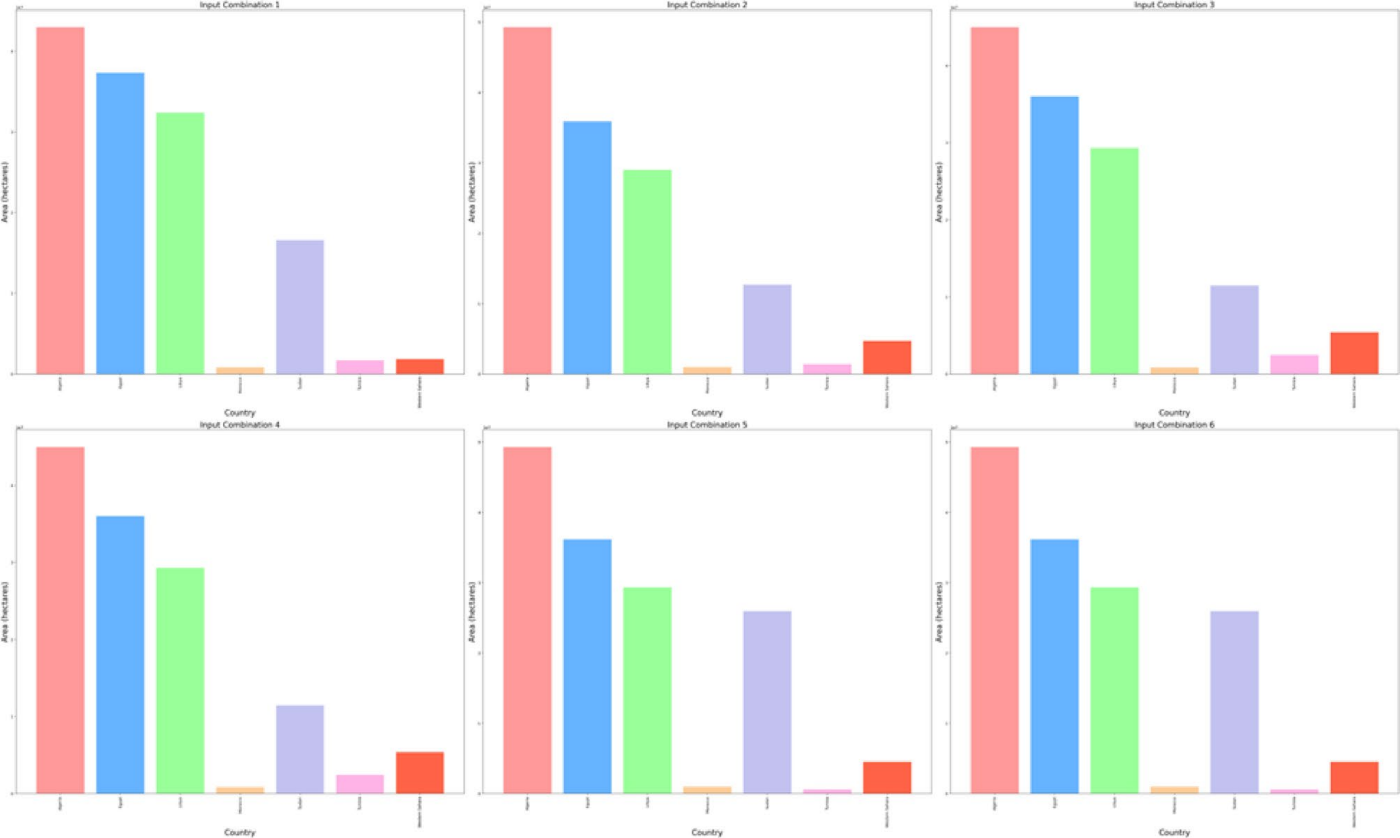
Area calculated in hectares for countries in the North Africa region for the highly suitable cluster for solar pond development site selection for six input combinations.

In the initial phase, North Africa, comprising seven countries, namely, Egypt, Sudan, Libya, Tunisia, Algeria, Morocco, and the Western Desert, was the focus of the study as shown in Fig. 8. The application of DBSCAN clustering was executed for this selected region, with various input combinations tested. The first set of input parameters, considering Radiation direct normal radiation (DNR), resulted in the identification of four clusters: “noise”, “High suitability”, “moderate suitable”, and “low suitable”. The “High Suitable” cluster was identified across the entire region marked in red, covering a total area of 4.7 × 10^8^ hectares, primarily distributed in Algeria, Egypt, Libya, and Sudan as shown in Fig. 9 and Figure S4. The “Moderate Suitable” cluster was pinpointed for a multipolygonal object positioned along the northern coastline, extending from the Mediterranean Sea and North Atlantic Ocean to the Red Sea. The multipolygonal region spans coordinates of 0.25°W and 35.25°N to 7.75°E and 36.75°N in Algeria; 12.25°E and 32.75°N to 22.75°E and 32.75°N in Libya; 8.75°E and 35.25°N to 11.25°E and 34.75°N in Tunisia; 27.25°E and 31.25°N to 35.25°E and 24.25°N in Egypt; and 16.25°W and 27.25°N in the Western Desert. Additionally, coordinates of 10.25°W and 28.75°N were obtained for Morocco, and 24.75°E and 9.75°N were obtained for 36.25°E and 22.25°N for Sudan. The total area of the multiple polygons was calculated to be 2.6 × 10^6^ hectares. The “Low Suitable” cluster was identified with coordinates ranging from 1.25°E and 36.25°N to 8.25°E and 36.75°N in Algeria; 14.75°E and 32.25°N to 15.25°E and 31.25°N in Libya; 8.75°E and 36.25°N to 9.75°E and 31.25°N in Tunisia; and 25.25°E and 31.25°N to 26.75°E and 31.25°N in Egypt, as well as 13.25°W and 26.75°N to 15.25°W and 23.25°N in the Western Desert. The area of the multipolygonal region for this cluster was calculated to be 1.5 × 10^6^ hectares. In the second input combination, which considered both DNR and temperature, 4 clusters were identified. One cluster, designated as “High Suitable,” encompasses a total area of 4.7 × 10^8^ hectares, primarily extending into the countries in the following order: Algeria, Egypt, Libya, and Sudan. Another one labelled as “Moderate Suitable”, spans from 3.25°E and 36.25°N to 3.75°E and 36.25°N in Algeria, covering an area of 456.6 × 10^2^ hectares. Another cluster, labelled “Low Suitable”, ranges from 4.25°E and 36.25°N to 6.75°E and 36.25°N in Algeria, with an area of 69.9 × 10^2^ hectares.

When considering the third input combination, which included DNR, temperature, and wind speed, 4 clusters were also identified. One cluster is termed as “High Suitable” has an area of 4.7 × 10^8^ hectares spread over Algeria, Egypt, Libya and Sudan. The “Moderate Suitable” cluster’s coordinates span from 0.25°W and 35.25°N to 8.25°E and 36.75°N in Algeria; 21.25°E and 32.25°N to 22.25°E and 32.25°N in Libya; and 8.75°E and 36.25°N to 9.75°E and 36.75°N in Tunisia. The area of the multipolygonal region has a value of 4.3 × 10^6^ hectares. Another cluster termed “Low Suitable” features a multipolygon area with coordinates of 3.75°E and 36.25°N to 8.25°E and 36.25°N in Algeria with an area value of 1.2 × 10^5^ hectares.

When evaluating the fourth input combination, 4 clusters were identified. One cluster termed as “High Suitable” spread over countries include Algeria, Egypt, Libya and Sudan has the same area as before. The other cluster, labeled “Moderate Suitable”, delineates a multipolygon region with coordinates and area values nearly identical to those obtained from the third input combination. This finding suggested that the categorization of clusters remains unaffected by the inclusion of the clear sky percentage in the input combination.

Considering the cloud cover in the fifth input combination, the analysis also yields 4 clusters, resulting in different outcomes for the clusters suitable for solar pond development. One cluster termed as “High Suitable” has an area of 4.3 × 10^8^ hectares spread over countries in Algeria, Egypt, Libya and Sudan. A cluster referred to as “Moderate Suitable” explored an area with coordinates ranging from 0.25°E and 33.25°N to 1.75°E and 33.75°N in Algeria, covering an area of 2.2 × 10^5^ hectares. Another cluster, termed “Low Suitable,” encompasses an area of 4.4 × 10^7^ hectares in Sudan, with coordinates of 23.75°E and 10.25°N to 34.25°E and 10.25°N.

Finally, when factoring in the precipitation data in the sixth input combination, the analysis again yielded the same 4 clusters. The data reveal multipolygonal areas for “High Suitable” cluster have an area like before. Other cluster is termed as the “Moderate Suitable” cluster, and the “Low Suitable” cluster shares the same coordinates and area values. This phenomenon is attributed to the minimal influence of precipitation on the cluster analysis over North Africa, given the nearly zero values observed over a 20-year period.

The results indicate an increase in epsilon values as indicated in Figure S3 and adapted to values in Table S1 as more parameters are considered in the input combinations. Specifically, the epsilon values increase from 0.048 when considering only DNR to 0.48 (a tenfold increase) when temperature is included in the input combination. This value remains consistent at 0.48 after considering the wind speed but increases to 1.78 (more than threefold) when the clear sky parameter is considered and to 1.8 when precipitation is factored in. These variations are reflected in significantly larger dimensions observed when the temperature parameter is included in the input combination than when considering only the DNR. This difference can be attributed to the weak correlation between DNR and temperature, as evidenced by the correlation value of 0.09 in Figure S2. In contrast, the suitable cluster shows a much larger increase when the wind speed is included, with values ten thousand times and one thousand times greater than those obtained for the second input combination. The results indicate that DBSCAN is influenced not only by the degree of correlation between parameters but also by other measures that exclude outliers in the data. Similar results are observed for the areas of suitable locations in the fourth input combination, reflecting an unchanged epsilon value, while a decrease is observed when cloud cover is factored into the fifth input combination. This trend continues in the sixth input combination, with minimal changes in the epsilon value.

The differences in the application of DBSCAN clustering between a smaller dataset in North Africa and a larger dataset covering the entire continent of Africa are evident. The increased variability of the data across continents makes it challenging to expand the clusters into suitable ones, as it becomes difficult to assign more points within the epsilon value that share the same characteristics or have high correlation with neighboring points. This distinction became apparent when applying the clustering approach in North Africa, where more suitable areas were distinguished that did not appear when the application was performed across the entire African continent.

**Fig. 10:**
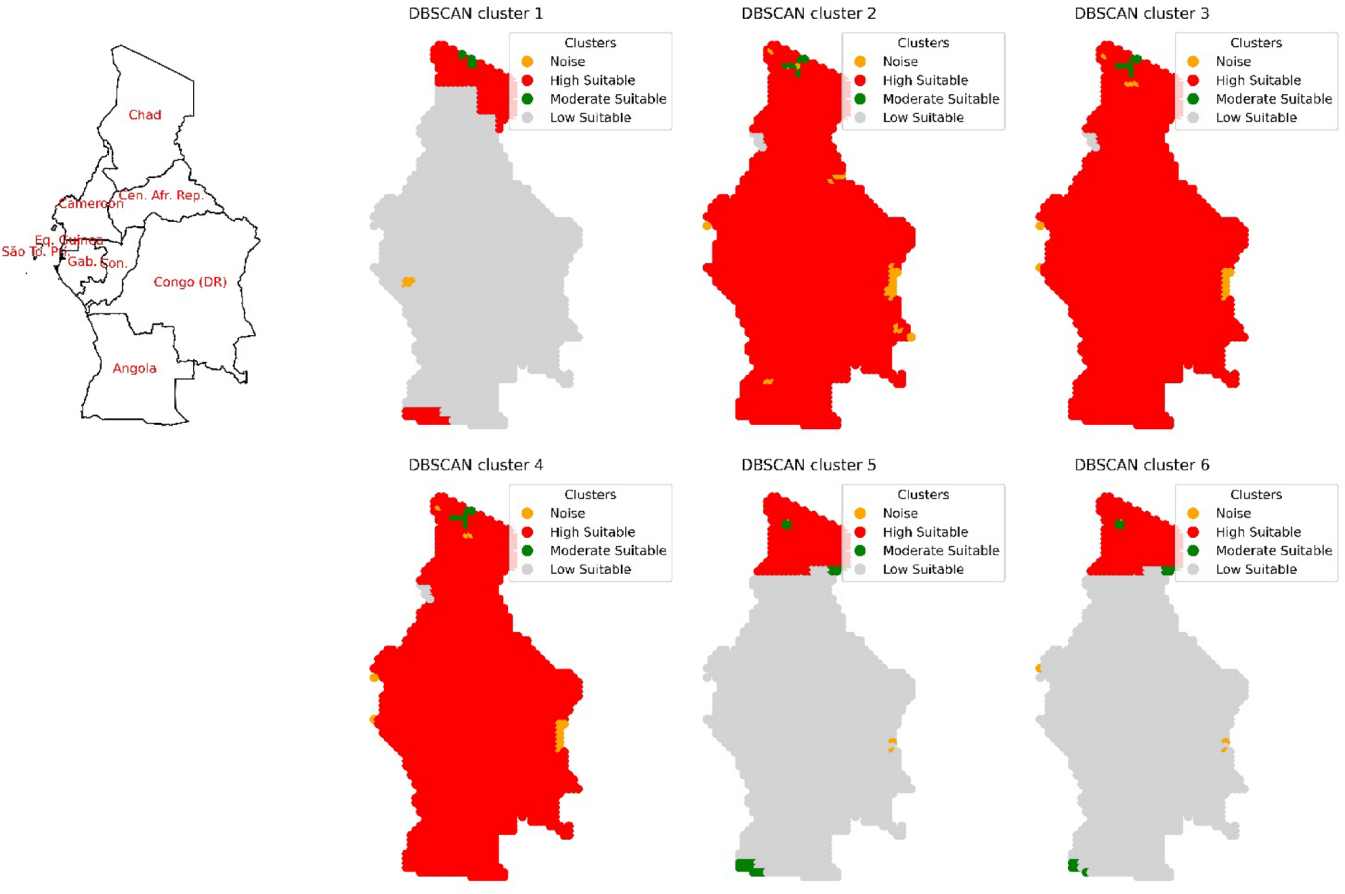
The spatial DBSCAN cluster outputs distributions of different combinations of environmental conditions across the geographical region of Central Africa.

**Fig. 11:**
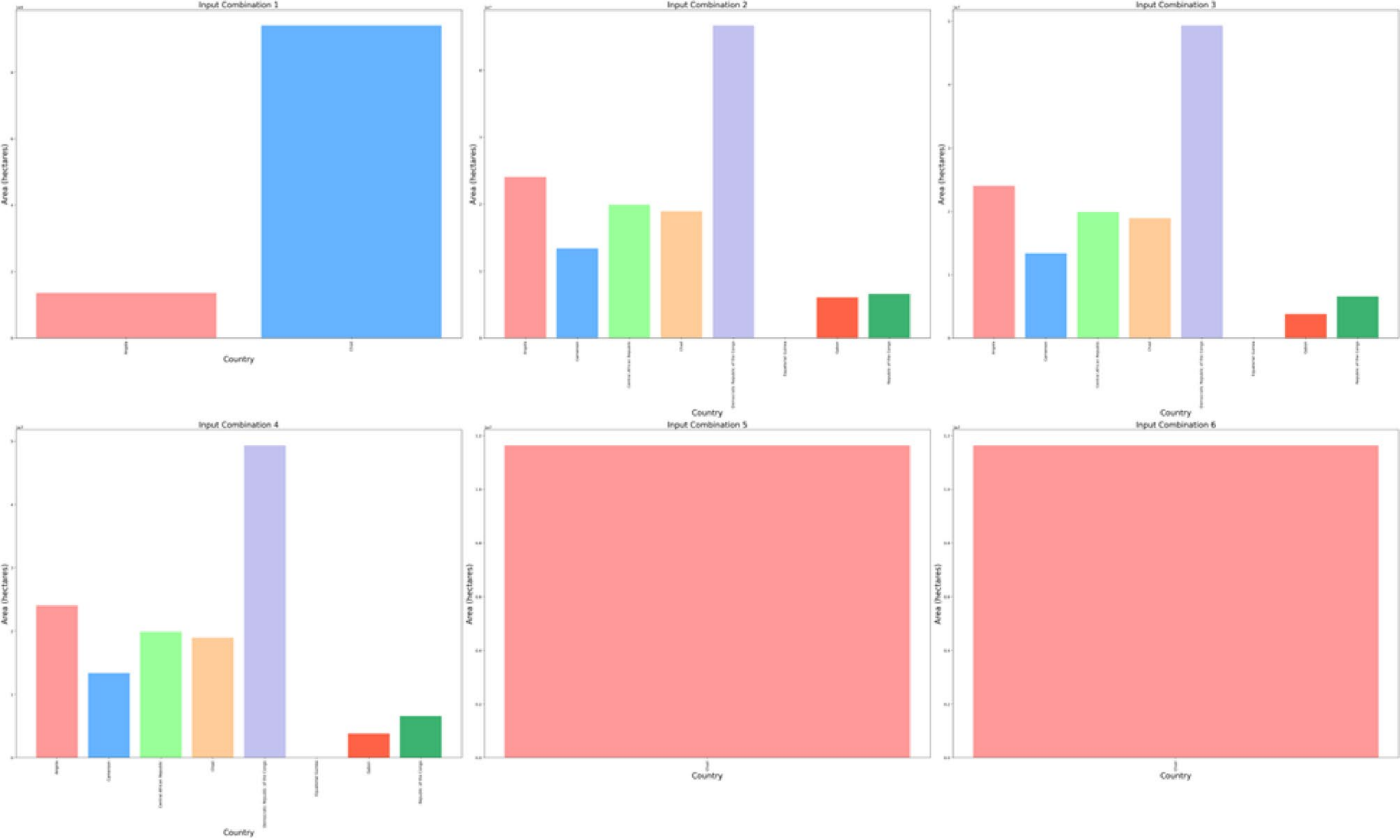
Area calculated in hectares for countries in Central Africa for the cluster of high suitability for site selection of solar ponds development.

Central Africa comprises 11 countries, namely, Angola, Burundi, Chad, Equatorial Guinea, Gabon, Cameroon, the Central African Republic, the Democratic Republic of Congo, the Republic of Congo, Rwanda, and São Tomé and Principe. The application of DBSCAN clustering was used to sort environmental remote sensing data into clusters based on various input combinations, as shown in Fig. 10. After considering the first input combination, four clusters were obtained: "Noise," "High Suitable," "Moderate Suitable," and "Low Suitable." A cluster termed as “High Suitable” has an area of 1.88 × 10^8^ hectares occupied in Chad mainly then Angola. The “moderate Suitable” cluster spans coordinate from 18.25°E and 22.25°N to 19.75°E and 21.75°N in Chad, covering an area of 2.7 × 10^6^ hectares. Moreover, the “low Suitable” cluster encompasses coordinates in Chad, Cameroon, the Central Africa Republic, Equatorial Guinea, Gabon, the Republic of Congo, the Democratic Republic of Congo, and Angola, as shown in Fig. 11 and Figure S7 with a multipolygonal object covering an area of 4.7 × 10^8^ hectares. With the second input combination, considering temperature and DNR, four clusters were obtained. A cluster termed as “High Suitable” created a multipolygon with an area of 6.5 × 10^8^ hectares spread in Democratic Republic of Congo. Angola, Central Africa Republic and Chad. The “Moderate Suitable” cluster creates a multipolygon with coordinates of 17.25°E and 21.25°N to 19.25°E and 21.75°N in Chad, covering an area of 1.2 × 10^6^ hectares. The “Low Suitable” cluster forms a region of coordinates in Chad and Cameroon, with an area of 4.4 × 10^6^ hectares.

These results demonstrate the application of DBSCAN clustering to analyze environmental remote sensing data in Central Africa, providing valuable insights into suitable and highly suitable areas within the region.

For the third input combination, four clusters were identified. One cluster termed as “High Suitable” was created an area for a multipolygonal with a value of 6.47 × 10^8^ hectares. The cluster labeled “Moderate Suitable” encompasses the coordinates in Chad ranging from 17.25°E and 21.25°N to 18.75°E and 20.25°N, covering an area of 9.3 × 10^6^ hectares. Another cluster, “Low Suitable”, shares almost identical coordinates as the previous input combination, following the application of wind speed in conjunction with temperature and DNR.

In the case of the fourth input combination, the “High Suitable”, “Moderate Suitable” and “Low Suitable” clusters yielded consistent coordinates and area values, suggesting that the application of clear skies had a minimal impact on the clustering analysis.

The fifth input combination has a significant influence on the clustering analysis when the cloud amount is factored in. One cluster termed as “High Suitable” was set as a multipolygonal with an area of 10.14 × 10^7^ hectares in Chad. A cluster termed “Moderate Suitable” extends from 17.25°E and 20.25°N to 23.75°E and 15.75°N in Chad and from 12.25°E and 16.25°S to 14.75°E and 17.25°S in Angola, covering an area of 9.3 × 10^6^ hectares. Additionally, a “Low Suitable” cluster spans coordinates in Chad, Equatorial Guinea, Cameroon, and the Central African Republic, with a multipolygonal object reaching as far as the Democratic Republic of the Congo, Republic of Congo, Gabon, and Angola, encompassing an area of 5.5 × 10^8^ hectares. After incorporating precipitation into the sixth input combination, similar results were obtained, with some adjustments leading to a modification in the area for “High Suitable” cluster of a polygon area of 7.14 × 10^7^ hectares in Chad. Where the area of the “Low Suitable” cluster, resulting in an area of 5.8 × 10^8^ hectares.

The epsilon values demonstrate a similar pattern to that observed in North Africa as shown in Figure S5 and altered as indicated in Table S1. When considering only the DNR for the clustering analysis, an epsilon value of 0.08 was obtained. An increase in the epsilon value to 0.4 (five times more) resulted in high change, particularly in the highly suitable area for solar pond development. This change aligns with the higher correlation coefficient (0.1) between the DNR and temperature, as depicted in Figure S5, which is ten times greater than that obtained for the North Sea region. A slight increase in the epsilon value occurred when considering the wind speed parameter in the input combination, reaching a value of 0.68 with a correlation coefficient of 0.88 with the DNR. The trend remained consistent when considering clear sky data in the input combination, like the pattern observed in North Africa, and this was also reflected inversely in the area values for suitable areas for solar pond development, with a high correlation coefficient of 0.89 with the DNR. Additionally, the epsilon values increased threefold when considering the percentage of cloud cover, reaching a value of 2.2, leading to an increase in the suitable area with a negative correlation with DNR (-0.93). There was almost a slight increase in the epsilon value to 2.3 after considering the precipitation parameter, which was also reflected inversely in the suitable area, with an almost similar correlation coefficient with DNR (-0.88).

**Fig. 12:**
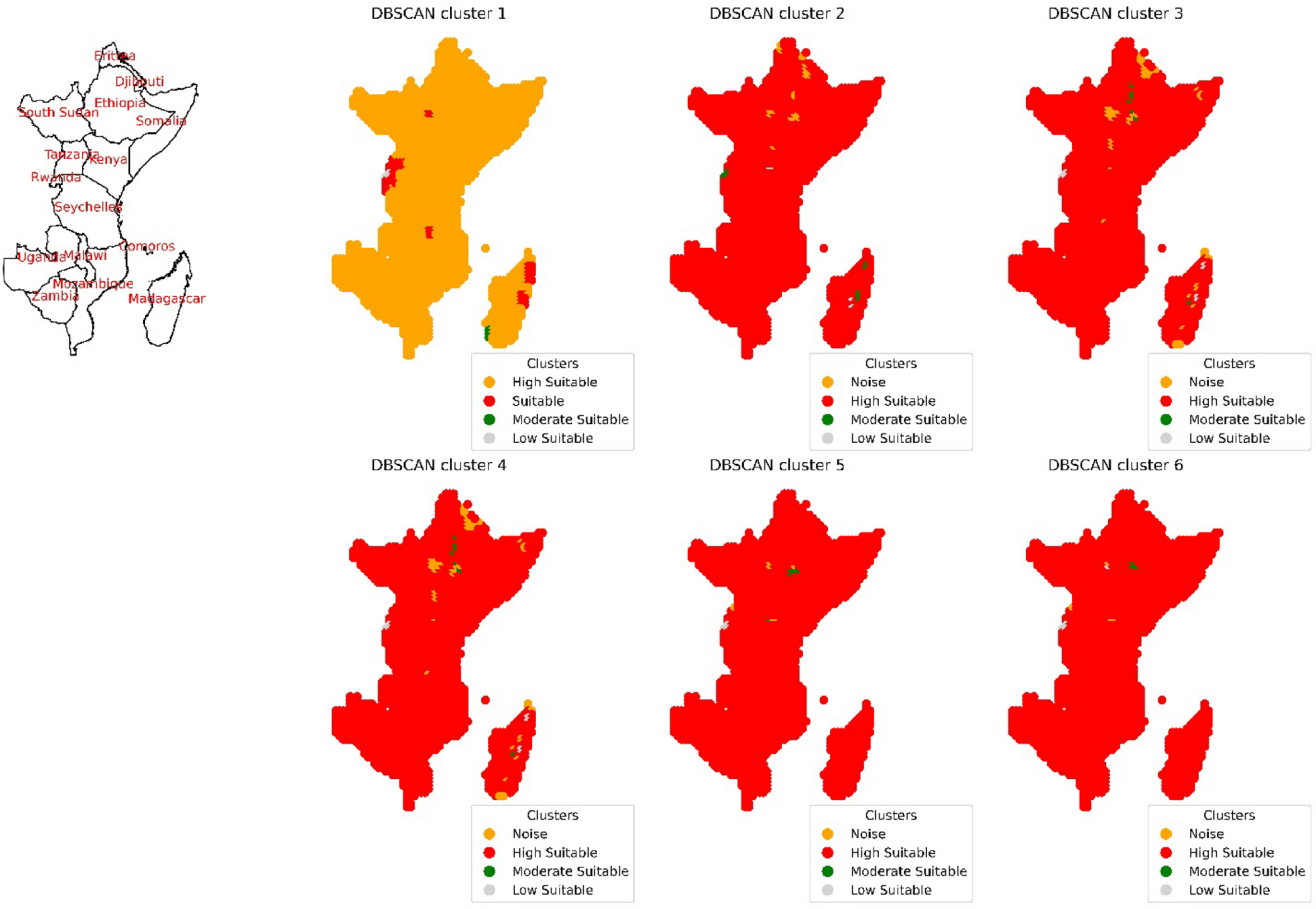
Spatial distribution of the DBSCAN cluster outputs after the application of different combinations of environmental conditions in the East Africa region.

**Fig. 13:**
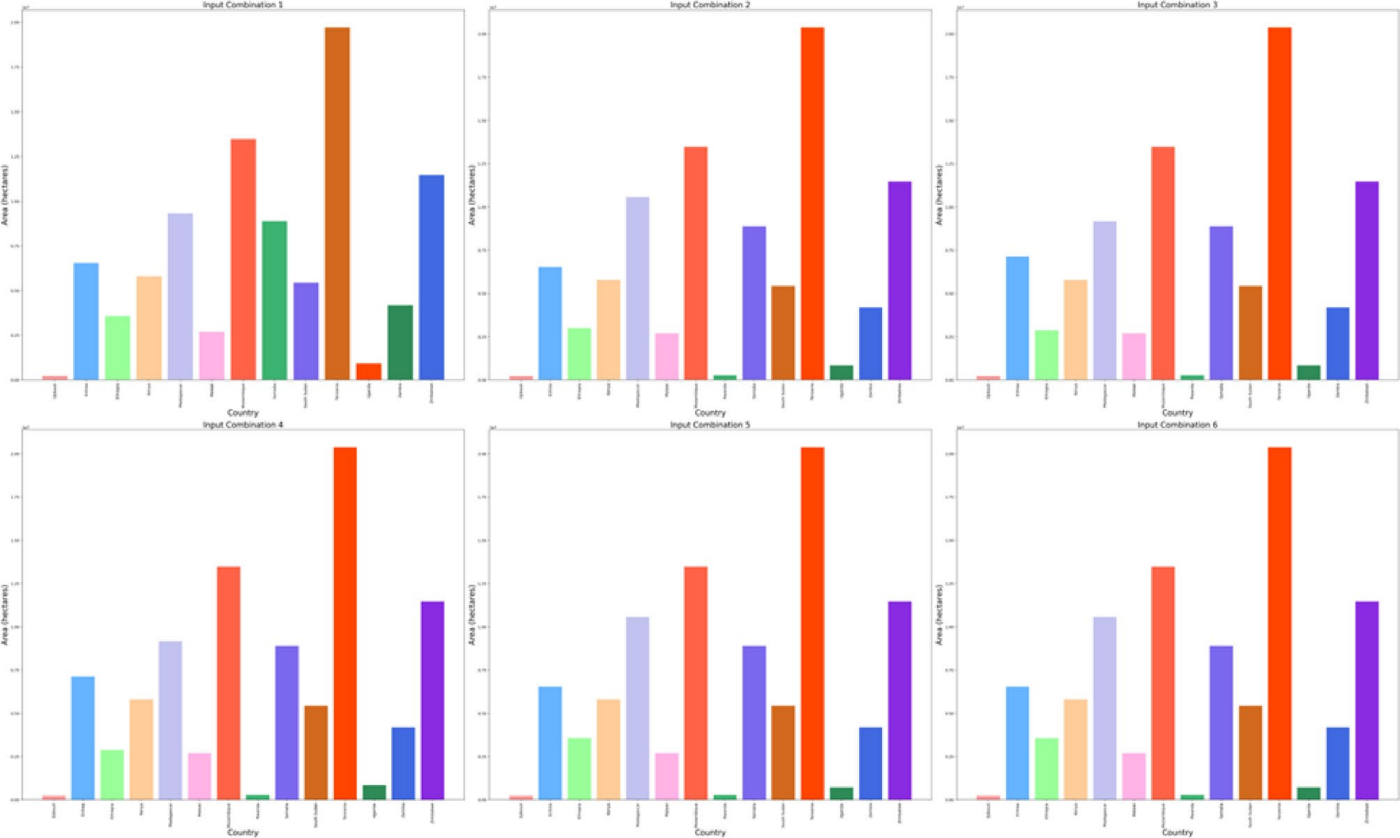
The area calculated in hectares for the highly suitable clusters for solar ponds site selection for countries in East Africa region for six input combinations.

The East African region encompasses Kenya, Rwanda, Uganda, Burundi, Tanzania, Djibouti, Somalia, Comoros, South Sudan, Madagascar, Malawi, Zambia, Zimbabwe, Mozambique, Mayotte, and Ethiopia. The utilization of DBSCAN clustering with various input combinations yields distinct clusters, as shown in Fig. 12. Following the implementation of the initial input combination, four clusters emerged: "High Suitable," "Suitable," "Moderate Suitable," and “Low Suitable”. A cluster termed as “High Suitable” create a multipolygon primarily in Tanzania, Mozambique, Zimbabwe and Somalia as shown in Fig. 13 and Figure S10 with an area of 5.87 × 10^8^ hectares. Within the “Suitable” cluster, multipolygonal objects are situated within coordinates spanning from 48.25°E and 14.25°S to 48.75°E and 19.75°S in Madagascar; 35.25°E and 7.25°N to 35.75°E and 7.75°N in Ethiopia; and 29.75°E and 0.75°S to 31.75°E and 0.75°N in Uganda. The multipolygonal object spans from 29.25°E and 2.75°S to 30.75°E and 2.25°S in Rwanda, from 29.25°E and 2.75°S to 29.75°E and 3.75°S in Burundi, and from 30.75°E and 1.25°S to 35.75°E and 9.75°S in Tanzania, with an area value of 1.9 × 10^6^ hectares. Additionally, a cluster labeled “Moderate Suitable” occupies a region with coordinates of 43.75°E and 23.25°S to 43.75°E and 24.25°S in Madagascar, encompassing an area of 24.6 × 10^2^ hectares. Another cluster denoted “Low Suitable”, has coordinates of 29.75°E and 1.25°S to 29.75°E and 1.75°S in Uganda and Rwanda, respectively, with an area value of 2.2 × 10^5^ hectares. Following the application of the second set of inputs, four clusters were identified: "Noise," "High Suitable," "Moderate Suitable," and "Low Suitable." Among these, one cluster termed as “High Suitable” create a multipolygon with an area of 5.18 × 10^8^ hectares mainly in Tanzania, Mozambique and Zimbabwe. the “Moderate Suitable” cluster forms a multipolygonal object with coordinates spanning from 49.25°E and 14.25°S to 48.25°E and 19.25°S in Madagascar and from 29.75°E and 1.25°S to 29.75°E and 1.75°S in Uganda and Rwanda, covering an area of 6.3 × 10^7^ hectares. Additionally, the “Low Suitable” cluster encompasses an area with coordinates of 47.25°E and 20.25°S to 47.25°E and 19.25°S in Madagascar and from 35.75°E and 0.75°S to 36.25°E and 0.75°S in Kenya, with a total area of 8.6 × 10^6^ hectares. After the application of the subsequent third input combination, 4 clusters, as before, were obtained with an epsilon value of 0.6. One cluster termed as “High Suitable” create an area with a value of 5.82 × 10^8^ hectares primarily in Tanzania, Mozambique and Zimbabwe. The cluster termed “Moderate Suitable” has coordinates of 39.25°E and 10.75°N to 39.75°E and 6.75°S in Ethiopia. The area of a multipolygonal object has a value of 1.4 × 10^6^ hectares. The cluster denoted “Low Suitable” features a region with coordinates ranging from 49.25°E and 14.25°S to 48.25°E and 19.25°S in Madagascar and from 29.25°E and 1.25°S to 29.75°E and 1.75°S in Rwanda. The area of the multipolygon has a value of 6.2 × 10^6^ hectares. Similar results were achieved following the application of the subsequent fourth input combination. The inclusion of the clear sky parameter alongside DNR, temperature, and wind speed did not yield a significant impact. However, a reduction in the suitable area was observed upon factoring in the percentage of cloud cover. Specifically, a cluster termed as “High Suitable” create an area of 5.87 × 10^8^ hectares primarily in Tanzania, Mozambique and Zimbabwe. The other is the “Moderate Suitable” cluster exhibited an area of 2.5 × 10^6^ hectares, with the multipolygonal object spanning from 38.75°E and 6.25°N to 39.75°E and 6.75°N in Ethiopia and from 35.75°E and 0.25°S to 35.75°E and 0.75°S in Kenya. Similarly, the “Low Suitable” cluster had an area of 2.2 × 10^5^ hectares, encompassing a multipolygonal region with coordinates of 29.75°E and 1.25°S to 29.75°E and 1.75°S in Uganda and Rwanda, respectively. Subsequently, upon considering precipitation in the sixth input combination, four clusters were identified. Notably, A cluster termed as “High Suitable” has an area of almost the same value as before also primarily in Tanzania, Mozambique and Zimbabwe. The “Moderate Suitable” cluster did not delineate any specific region, while the “Low Suitable” cluster delineated a region with an area of 2.4 × 10^6^ hectares, with coordinates ranging from 29.75°E and 1.25°S to 29.75°E and 1.75°S in Uganda and Rwanda, respectively.

In the East Africa region, the epsilon values follow a similar trend to those observed in the district regions after the application of different parameters in the same order within the input combinations. Those values were shown in Figure S9 and altered as indicated in Table S1. An epsilon value of 0.08 was indicated after considering DNR in the input combinations, which increased fivefold to a value of 0.385 after including temperature in the input combination with a correlation coefficient of 0.07 with DNR. As in previous district regions, there was a slight increase in epsilon values after considering wind speed data, resulting in a value of 0.6, similar to that obtained after considering the dimensionless clear sky parameter, with correlation coefficients of 0.47 and 0.26 between wind speed and clear sky conditions with DNR, respectively as shown in Figure S8. Additionally, a threefold increase in the DNR yielded an epsilon value of 1.8 after considering the cloud cover parameter in the input combinations, with a negative correlation coefficient with DNR (− 0.86). Similarly, for the previous district region, there was a slight increase in the epsilon value to 2.15 after considering precipitation in the input combinations, with a lower correlation coefficient with DNR (− 0.38).

**Fig. 14:**
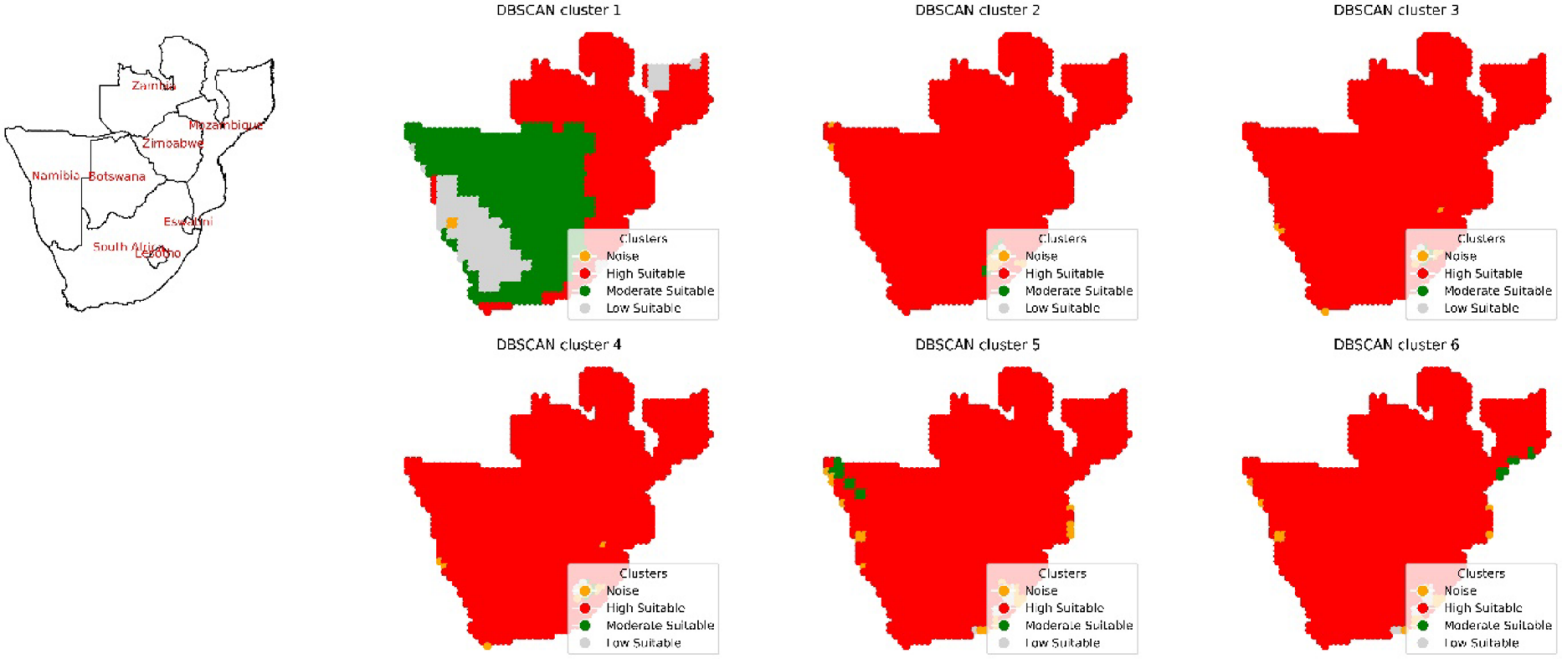
Overviews of the spatial distribution of the clusters categorized after the application of DBSCAN cluster analysis in the southern African region.

**Fig. 15:**
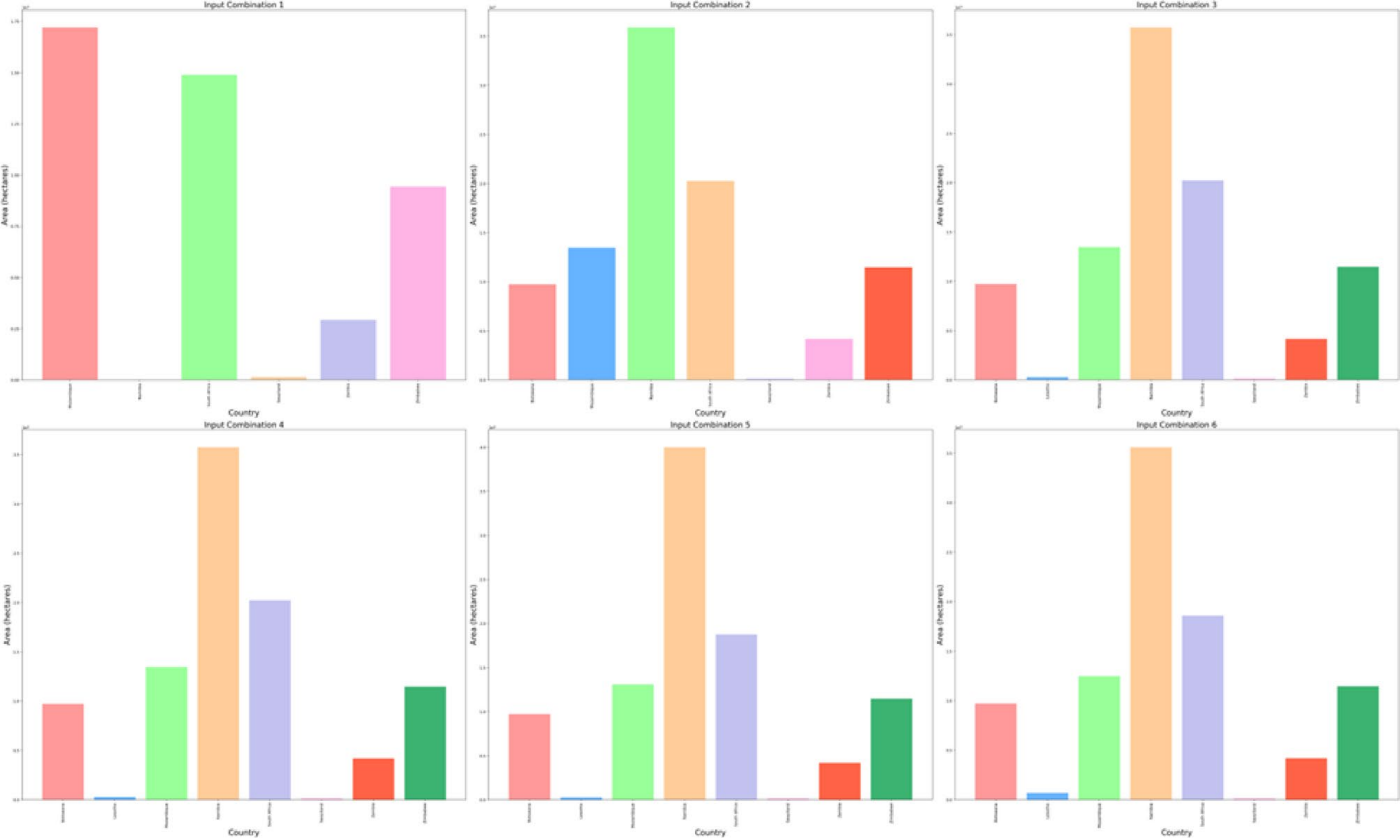
Area calculated in hectares for countries in Southern Africa for the high suitability cluster for solar ponds development site selection for six input combinations.

In the region of southern Africa, there are 8 regions: Botswana, Lesotho, Mozambique, Namibia, South Africa, Swaziland, Zambia, and Zimbabwe. The process of DBSCAN clustering was applied to a set of input combinations using environmental remote sensing data, resulting in the categorization of clusters, as shown in Fig. 14. For the first input combination, 4 clusters were identified and labeled “Noise”, “High Suitable”, “Moderate Suitable”, and “Low Suitable”. A cluster labeled “High Suitable” create a polygon with an area of 5.38 × 10^8^ hectares in Mozambique, South Africa and Zimbabwe as shown in Fig. 15 and Figure S13. The “Moderate Suitable” cluster formed a multipolygonal zone covering an area of 1.2 × 10^8^ hectares with coordinates ranging from 22.25°E and 18.25°S to 21.75°E and 26.75°S in Botswana; 28.25°E and 28.75°S to 27.25°E and 30.25°S in Lesotho; and 12.25°E and 17.25°S to 19.75°E and 24.75°S in Namibia. In South Africa, the coordinates spanned from 16.75°E and 28.75°S to 24.75°E and 33.75°S, from 23.25°E and 17.25°S to 25.75°E and 17.75°S in Zambia, and from 27.75°E and 17.25°S to 29.75°E and 19.75°S in Zimbabwe. The “Low Suitable” cluster included multipolygonal objects with coordinates ranging from 12.75°E and 19.25°S to 13.75°E and 21.25°S in Namibia and from 39.25°E and 11.25°S to 36.25°E and 13.75°S in Mozambique, covering an area of 2.2 × 10^7^ hectares. After considering the subsequent second input combination, 4 clusters were identified. One cluster termed as “High Suitable” was created a multipolygon primarily in Namibia, South Africa and Mozambique with an area with a value of 6.78 × 10^8^ hectares. A multipolygonal object was formed for the “Moderate Suitable” cluster, covering an area of 9.8 × 10^5^ hectares with coordinates ranging from 28.75°E and 28.25°S to 27.75°E and 31.25°S in South Africa and from 28.25°E and 28.75°S to 27.75°E and 29.25°S in Lesotho. Within the “Low Suitable” cluster, a multipolygon object was defined with an area of 3.7 × 10^5^ hectares, with coordinates in Lesotho from 28.75°E and 28.75°S to 28.75°E and 29.75°S. After considering the third input combination, 4 clusters were identified. One cluster termed as “High Suitable” was created a multipolygon primarily in Namibia, South Africa and Mozambique with an area of 6.89 × 10^8^ hectares. The “Moderate Suitable” cluster results in the creation of a multipolygonal object with an area of 1.7 × 10^5^ hectares and coordinates from 29.25°E and 28.75°S to 29.75°E and 29.75°S in South Africa. The cluster denoted as “High Suitable” has an area of 5.6 × 10^5^ hectares with coordinates ranging from 28.75°E and 28.75°S to 28.75°E and 29.75°S in Lesotho. After considering the fourth input combination, the same results were obtained after considering that the clear sky parameter had no influence on the clustering analysis for the site selection process. When considering the fifth input combination, the cloud cover was considered in combination with other environmentally remote sensing data, resulting in the identification of four clusters. A cluster called “High Suitable” create a multipolygon in Namibia, South Africa and Mozambique with an area of 6.7 × 10^8^ hectares. The “Moderate Suitable” cluster included a multipolygonal region covering an area of 7.9 × 10^6^ hectares with coordinates ranging from 13.25°E and 17.25°S to 15.75°E and 20.75°S in Namibia. Additionally, the “Low Suitable” cluster included a region with an area of 1.9 × 10^6^ hectares, with coordinates ranging from 29.75°E to 29.25°S to 26.25°E to 33.25°S in South Africa.

Upon considering the precipitation parameter in the sixth input combination, the area for the suitable clusters was determined. A multipolygon created for a cluster termed “High Suitable” in Namibia, South Africa and Mozambique with an area of 6.89 × 10^8^ hectares. The “Moderate Suitable” cluster covered an area of 3.3 × 10^5^ hectares with coordinates ranging from 39.25°E to 16.25°S to 36.25°E to 18.75°S in Mozambique. The cluster termed “Low Suitable” created a region with coordinates between 29.75°E and 28.75°S to 29.75°E and 29.75°S, with a multipolygon object area of 8.06 × 10^5^ hectares in South Africa.

The trend for the epsilon values in southern Africa remains consistent when considering different parameters in the input combinations, following a similar order as shown in Figure S12 and adapted as shown in Table S1. An epsilon value of 0.07 was obtained after considering DNR in the input combination, which increased sixfold when factoring in temperature, exhibiting a moderate negative correlation coefficient with DNR (-0.4), as depicted in the correlation matrix diagram in Figure S11. This resulted in a reduction in the suitable area by a factor of one thousand for the second input combination. Like in the previous African districts, there was a slight increase in the epsilon values when considering the wind speed parameter, with similar values obtained when considering the dimensionless clear sky parameter at 0.79. The suitable areas obtained after considering both parameters exhibited similar values, with similar correlation coefficients of 0.84 and 0.93 between the wind speed and clear-sky parameters with the DNR, respectively. The epsilon values doubled when considering the percentage of cloud cover, reaching a value of 1.4 and revealing a negative correlation with DNR, with a coefficient of − 0.94, resulting in a tenfold increase in the suitable area. An increased epsilon value of 1.8 was obtained after considering precipitation in the input combination, exhibiting a high negative correlation with DNR (-0.86).

**Fig. 16:**
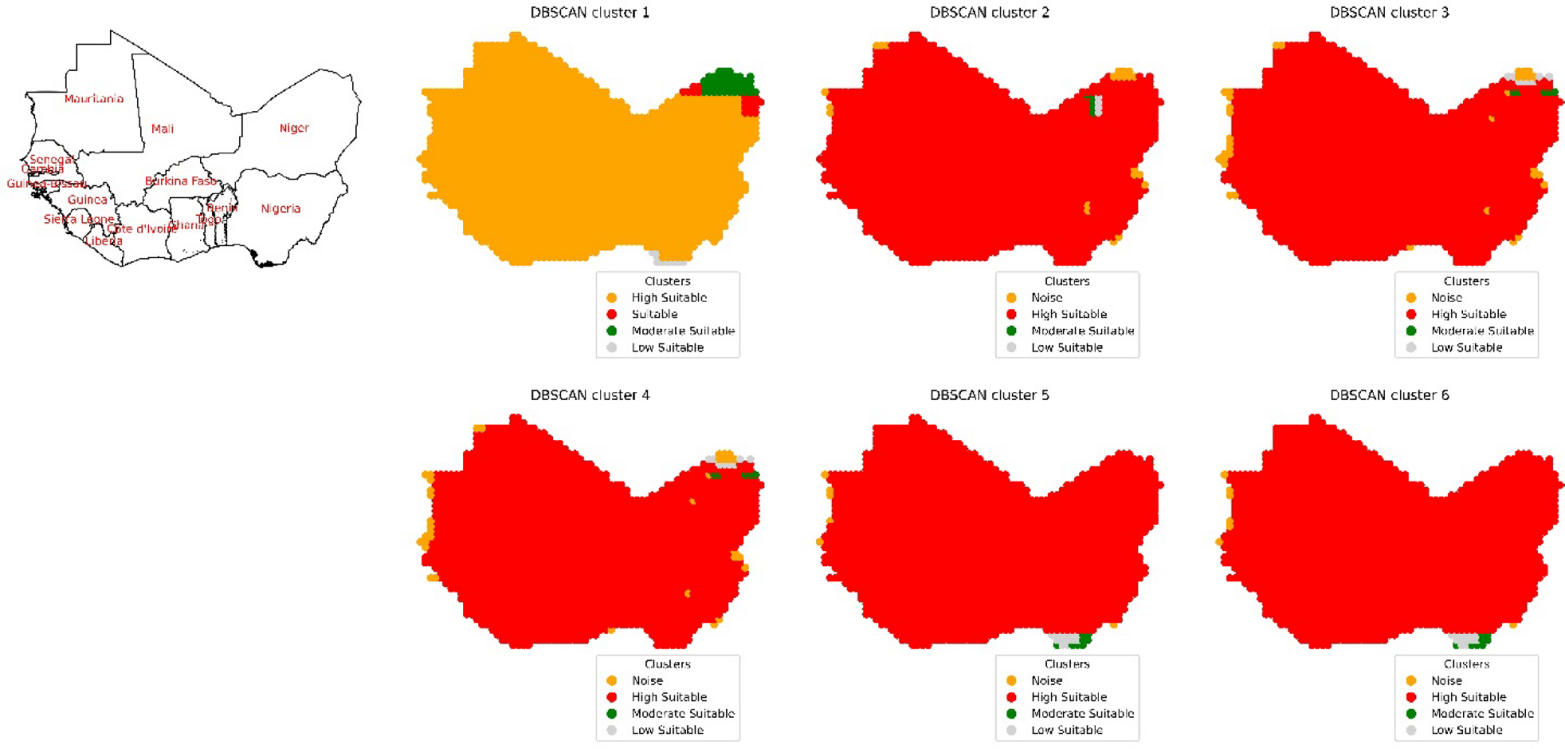
Spatial distribution of the clusters according to site suitability for solar pond development after the application of DBSCAN cluster analysis in the West Africa region.

**Fig. 17:**
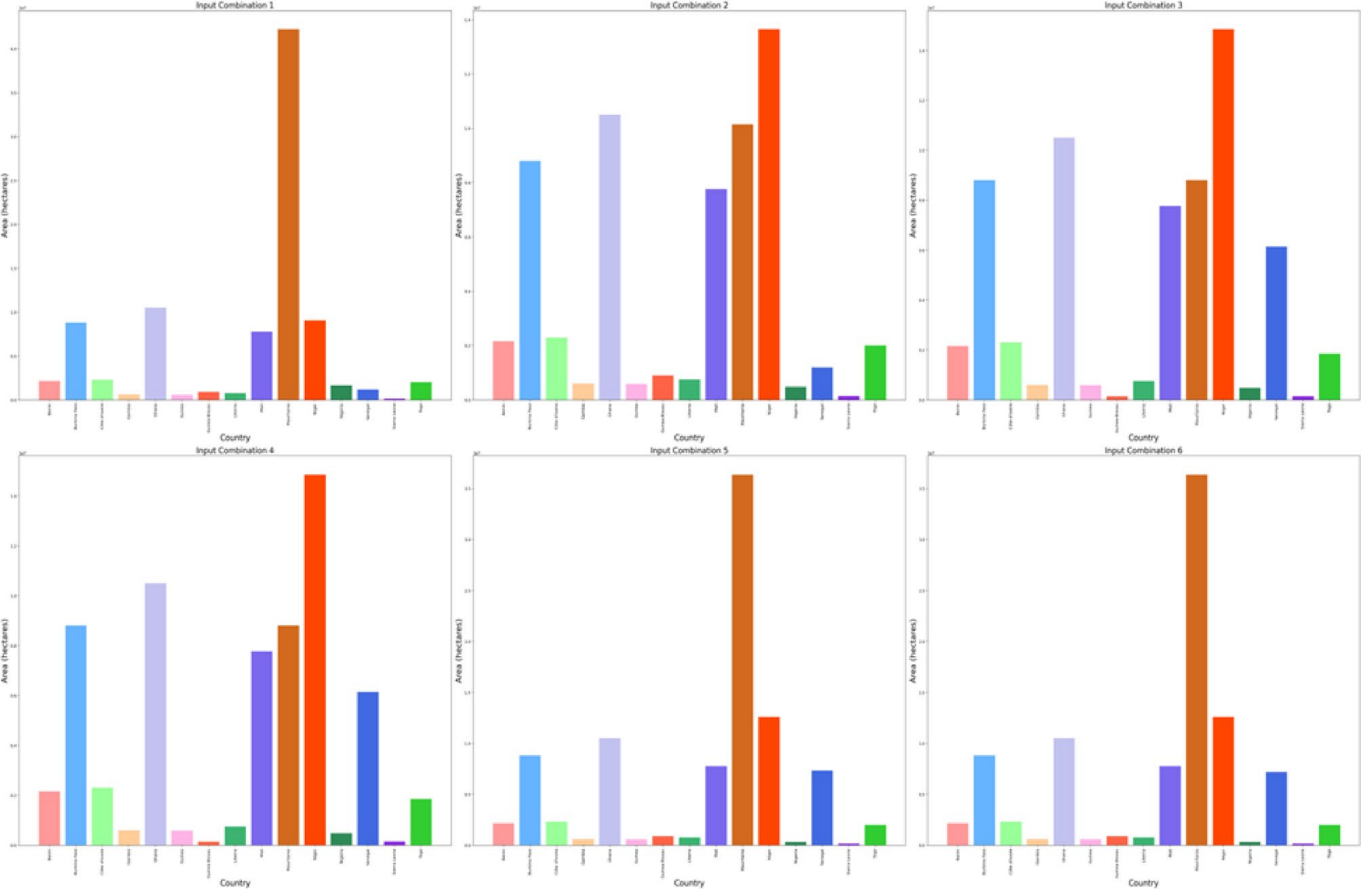
The calculated area in hectares for countries in West Africa for the highly suitable site selection for solar ponds development for six input combinations.

The procedure was repeated for the West Africa region, encompassing 16 countries: Benin, Burkina Faso, Cape Verde, Cote de Ivoire, Gambia, Ghana, Guinea, Guinea-Bissau, Liberia, Mali, Mauritania, Niger, Nigeria, Senegal, Sierra Leone, and Togo. DBSCAN clustering was applied, resulting in a consistent number of clusters determined by an assigned epsilon value, as shown in Fig. 16. After evaluating various combinations of environmental remote sensing data inputs, the first combination yielded 4 clusters labeled "High Suitable," "Suitable," "Moderate Suitable," and "Low Suitable." Where the suitable area and distribution of suitable areas over countries are shown in Fig. 17 and Figure S16. The “Moderate Suitable” cluster included multiple polygonal areas with coordinates ranging from 8.25°E and 21.25°N to 15.75°E and 20.25°S in Niger. The “Low Suitable” cluster encompassed coordinates from 10.25°E and 21.25°N to 12.75°E and 23.25°N in Niger, while the “Low Suitable” cluster spanned coordinates from 5.75°E and 4.75°N to 5.75°E and 5.75°N in Nigeria. Subsequently, a second input combination also resulted in 4 clusters: "Noise," "High Suitable," "Moderate Suitable," and "Low Suitable." The “Moderate Suitable” cluster covered coordinates from 9.25°E and 19.75°N to 9.25°E and 20.75°N in Niger, while the “Low Suitable” cluster spanned coordinates from 9.75°E and 19.25°N to 9.25°E and 20.75°N in Niger. This pattern continued with the third and fourth input combinations, each producing 4 clusters with distinct characteristics and coordinate ranges within Niger. When cloud cover was factored into the fifth input combination, 4 clusters were still obtained. The “Moderate Suitable” cluster formed an area with coordinates from 5.75°E and 4.75°N to 8.75°E and 5.75°N in Nigeria, while the “Low Suitable” cluster created a polygon with coordinates from 6.25°E and 4.75°N to 7.75°E and 5.75°N in Nigeria. Subsequently, the inclusion of precipitation as a parameter in the sixth input combination led to the creation of 4 clusters through the application of DBSCAN clustering, with similar patterns persisting.

Similarly, the West Africa region exhibited the same trend as shown in Figure S15 and modified according to the values in Table S1. An epsilon value of 0.09 was obtained after considering DNR in the input combination, which increased approximately threefold to 0.3 after factoring in temperature, showing a moderate positive correlation coefficient with DNR (0.27) as shown in Figure S14. There was also a slight increase in the epsilon value (0.43) after considering the wind speed parameter in the input combination, with a similar epsilon value obtained after considering the dimensionless clear sky parameter. The epsilon value increased threefold after considering the percentage of cloud cover, reaching a value of 1.5. The same value was obtained after considering the precipitation parameter in the input combination, which had an impact on the suitable area.

Overall, the utilization of AutoGIS processing integrated with DBSCAN clustering for solar pond development site selection reveals distinct patterns across Africa compared to those in the distracted African regions. The suitable patterns obtained after applying DBSCAN clustering for the district regions differed from those observed across the entire continent. The focus on the district regions aimed to evaluate the dataset’s variability for remotely sensed environmental data, which is critical for solar pond development. Similar trends were observed in the district African regions after considering DNR followed by temperature, resulting in an increase in the suitable area when wind speed was taken into account. However, across all the African districts, incorporating the dimensionless clear sky parameter did not alter the suitability of solar pond development sites. Conversely, the cloud cover percentage exhibited an increasing trend in terms of suitability for solar pond development sites. There was minimal variation when considering precipitation parameters, except in the central Africa region, which could be attributed to the distribution of zero values across the regions, except for Central Africa. Overall, these results underscore the significance of AutoGIS processing via the Python scripting language integrated with DBSCAN clustering for the site selection process, especially for large datasets such as the one we have for the African continent, comprising 18,000 data points across six remotely sensed environmental parameters.

## Conclusions

This study demonstrated the effective use of AutoGIS processing and DBSCAN clustering, supported by Python scripting, for identifying suitable sites for solar pond development across Africa using over 18,000 remotely sensed environmental data points. The analysis covered both the entire continent and its five subregions—North, Central, East, Southern, and West Africa. While regional variability influenced the clustering results, consistent patterns in suitable site distribution and epsilon values were observed across the subregions. Among the environmental factors, temperature showed limited correlation with Direct Normal Radiation (DNR), often reducing the area deemed suitable for solar ponds. This highlights the importance of integrating multiple parameters to enhance the accuracy of site selection. The practical deployment of these algorithms through user-friendly software tools supports broader accessibility, enabling informed decision-making even by non-experts. The findings are particularly relevant for regions with limited freshwater access and constrained investment in wastewater infrastructure, positioning solar pond technology as a viable, low-cost alternative. Ultimately, this approach supports the goals of the NEXUS framework by promoting sustainable solutions at the intersection of energy, water, and food security..

## Electronic supplementary material

Below is the link to the electronic supplementary material.

Supplementary Material 1

## Data Availability

The datasets used and analysed during the current study available from the corresponding author on reasonable request.
