# Supplementary material for: AutoGIS-driven solar pond site selection for water treatment in Africa aligned with the NEXUS framework
Source: Sci Rep. 2025 May 22;15:17743. doi: 10.1038/s41598-025-01778-6 (PMC12098993; doi:10.1038/s41598-025-01778-6)
Supplement: Supplementary file 1 — Supplementary Material 1 [file 41598_2025_1778_MOESM1_ESM.docx]

**AutoGIS-Driven Solar Pond Site Selection for Water Treatment in Africa Aligned with the NEXUS Framework**

Mahmoud Fatehy Altahan ^1,^*, Mohamed Nower ^2,^*

^1^ Central Laboratory for Environmental Quality Monitoring (CLEQM), National Water Research Center (NWRC), 13621 El-Qanater El-Khairia, Egypt.

^2^ Water Management Research Institute (WMRI), National Water Research Center (NWRC), 13621 El-Qanater El-Khairia, Egypt.

***** Correspondence: [mahmoud_abdalqader@nwrc.gov.eg](mailto:mahmoud_abdalqader@nwrc.gov.eg) (M.F.Altahan),

mohamed_nower@nwrc.gov.eg (M. Nower)

**Supplementary Information**

**Table S1.** Comparison between the epsilon values obtained from k-distance plots and adapted ones for Africa and five regional regions.

|  | **Africa** | | **North Africa** | | **Central Africa** | | **East Africa** | | **Southern Africa** | | **West Africa** | |
| --- | --- | --- | --- | --- | --- | --- | --- | --- | --- | --- | --- | --- |
| Input combination | k-distance plot | Adapted Ɛ | k-distance plot | Adapted Ɛ | k-distance plot | Adapted Ɛ | k-distance plot | Adapted Ɛ | k-distance plot | Adapted Ɛ | k-distance plot | Adapted Ɛ |
| 1^st^ inputs | 0.01 | 0.06 | 0.02 | 0.06 | 0.00 | 0.08 | 0.00 | 0.08 | 0.00 | 0.07 | 0.01 | 0.09 |
| 2^nd^ inputs | 0.44 | 0.90 | 0.31 | 0.90 | 0.36 | 0.4 | 0.31 | 0.365 | 0.31 | 0.45 | 0.24 | 0.3 |
| 3^rd^ inputs | 0.89 | 0.90 | 0.54 | 0.90 | 0.85 | 0.68 | 0.89 | 0.6 | 0.89 | 0.79 | 0.42 | 0.43 |
| 4^th^ inputs | 0.77 | 0.90 | 0.73 | 0.90 | 0.76 | 0.69 | 1.27 | 0.6 | 1.27 | 0.79 | 0.47 | 0.43 |
| 5^th^ inputs | 1.48 | 1.40 | 1.73 | 1.4 | 1.91 | 2.2 | 2.50 | 1.8 | 2.50 | 1.4 | 1.08 | 1.5 |
| 6^th^ inputs | 2.01 | 2.00 | 2.39 | 2.00 | 2.76 | 2.3 | 2.18 | 2.15 | 2.18 | 1.8 | 1.19 | 1.5 |


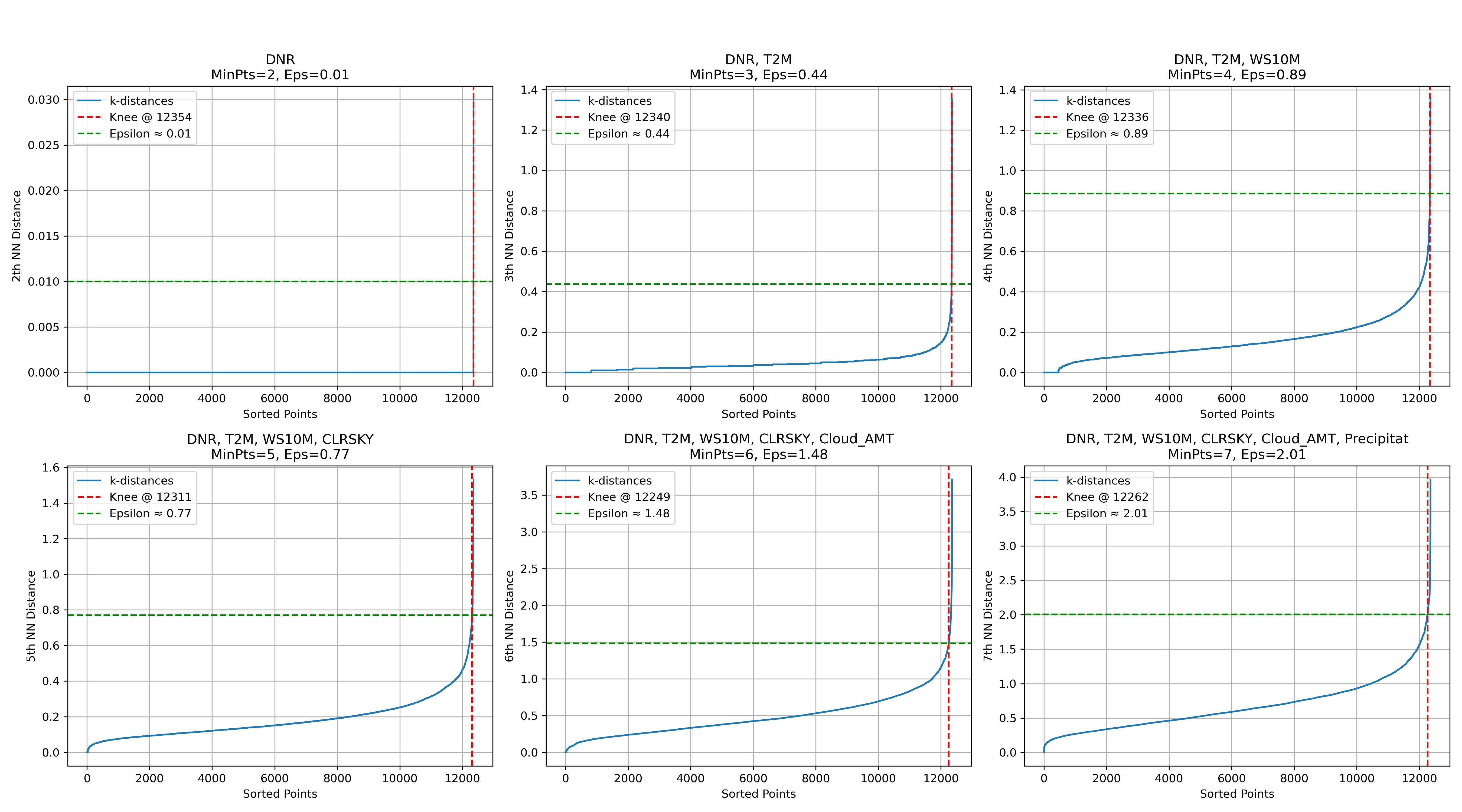


**Figure S1**. K-distance plots with minimum points and estimate epsilon values for six input combinations for the whole Africa.


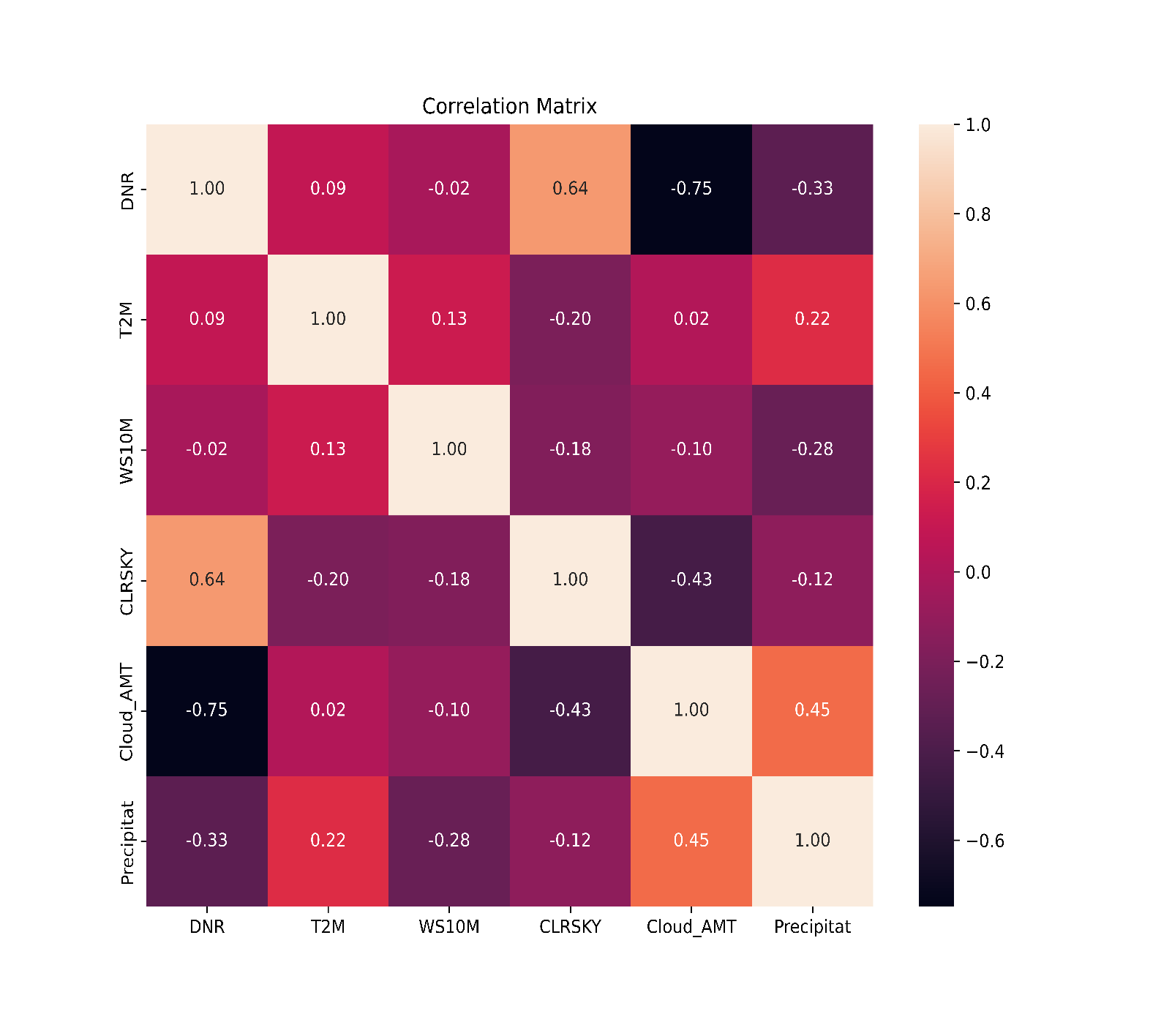


**Figure S2.** Correlation matrix for the environmental remote sensing data for North Africa.


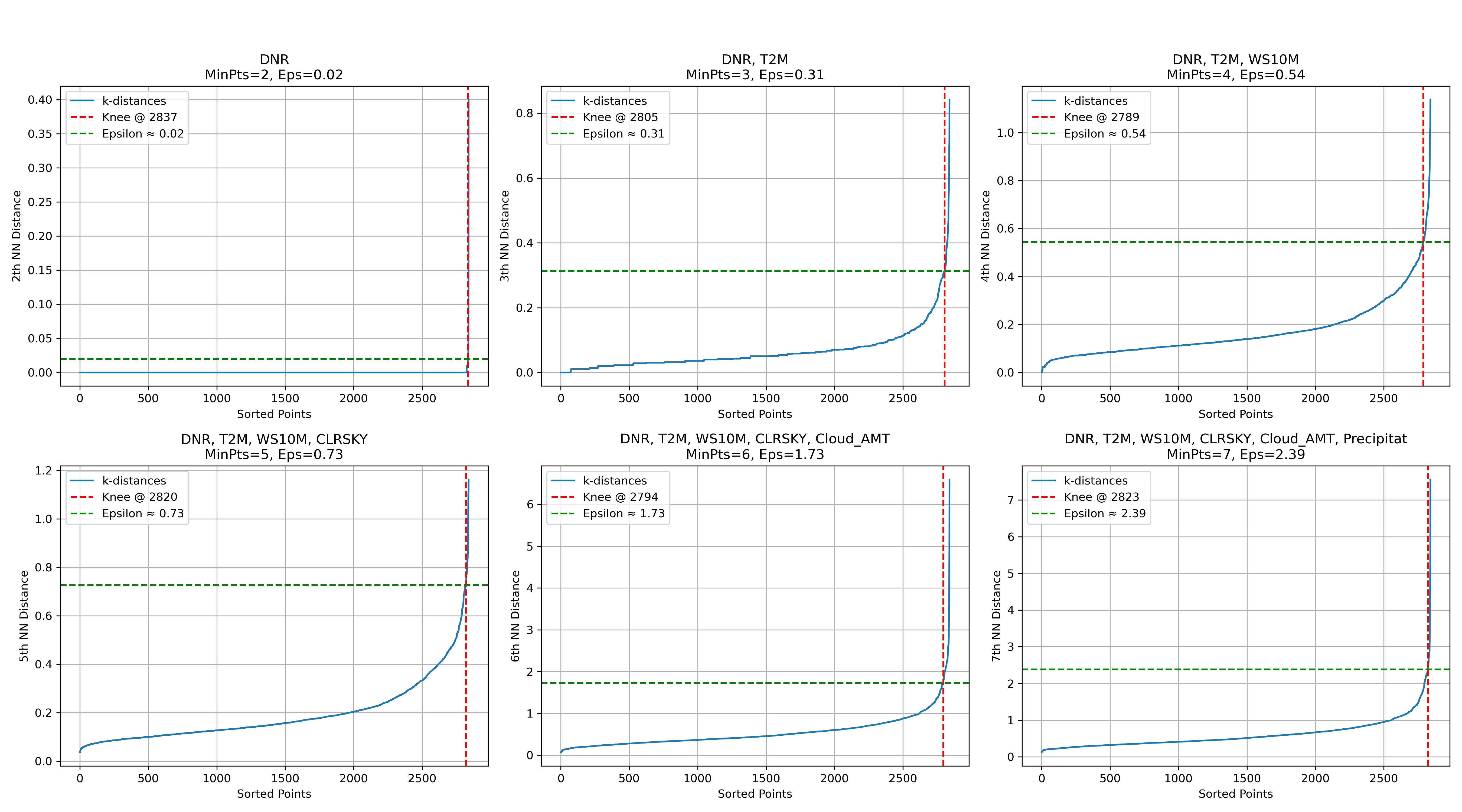


**Figure S3**. K-distance plots with minimum points and estimate epsilon values for six input combinations for North Africa.





**Figure S4**. Pie charts for the distribution of the percentages for the calculates areas in hectares for countries in the North Africa region for high suitable sites for solar ponds development for six input combination from left to right and from top to bottom.


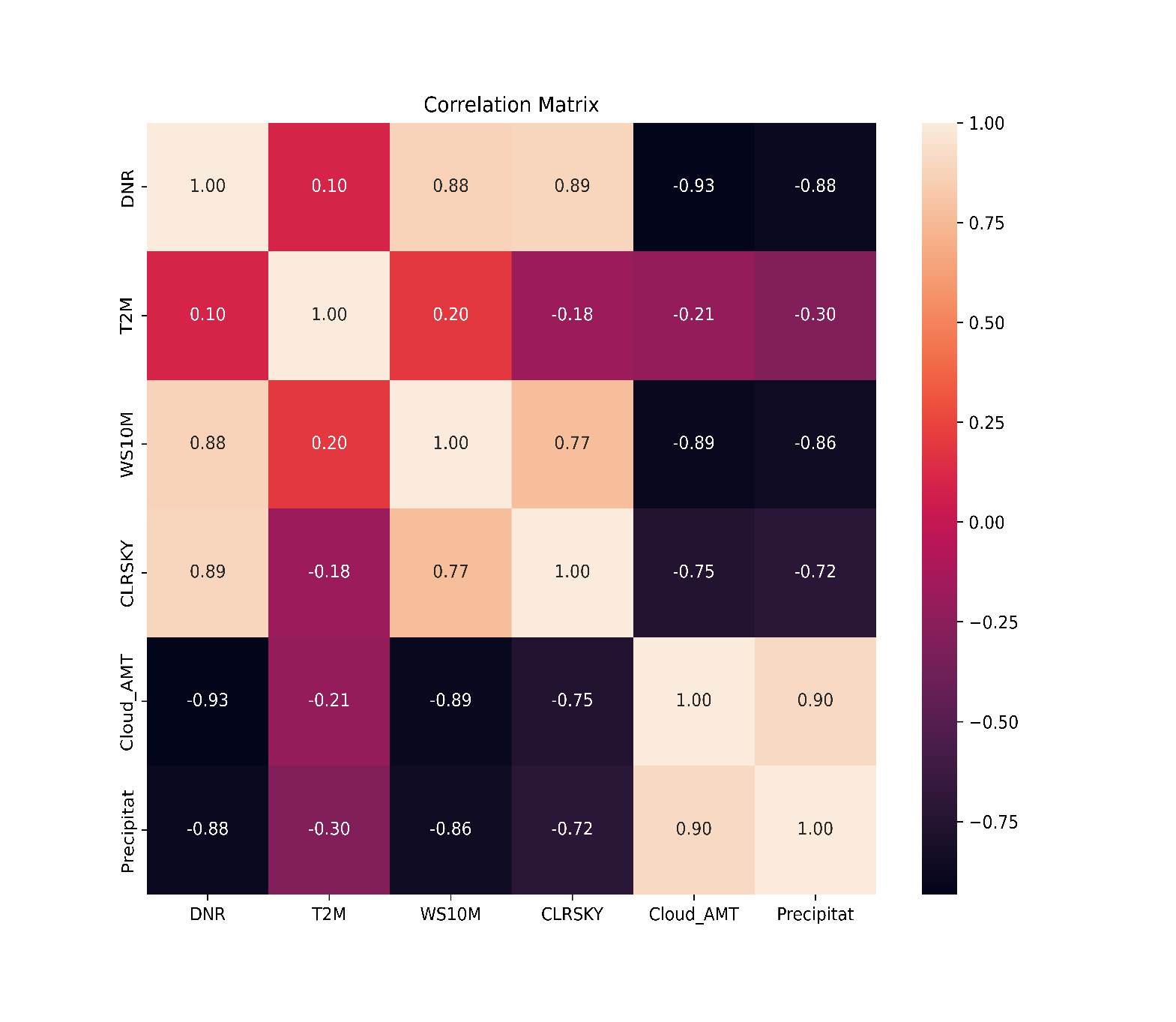


**Figure S5.** Correlation matrix for environmental remote sensing data for Central Africa.


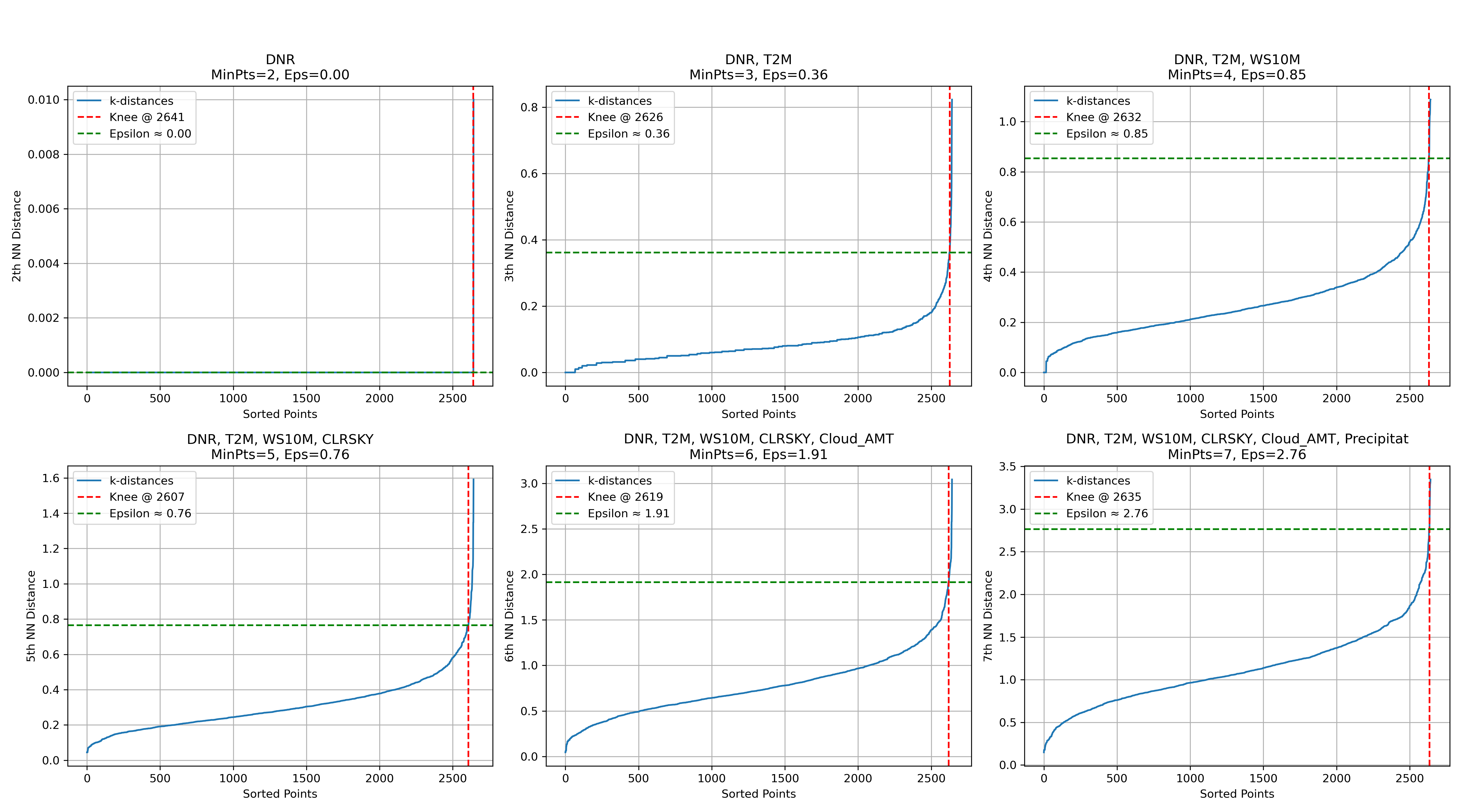


**Figure S6**. K-distance plots with minimum points and estimate epsilon values for six input combinations for Central Africa.


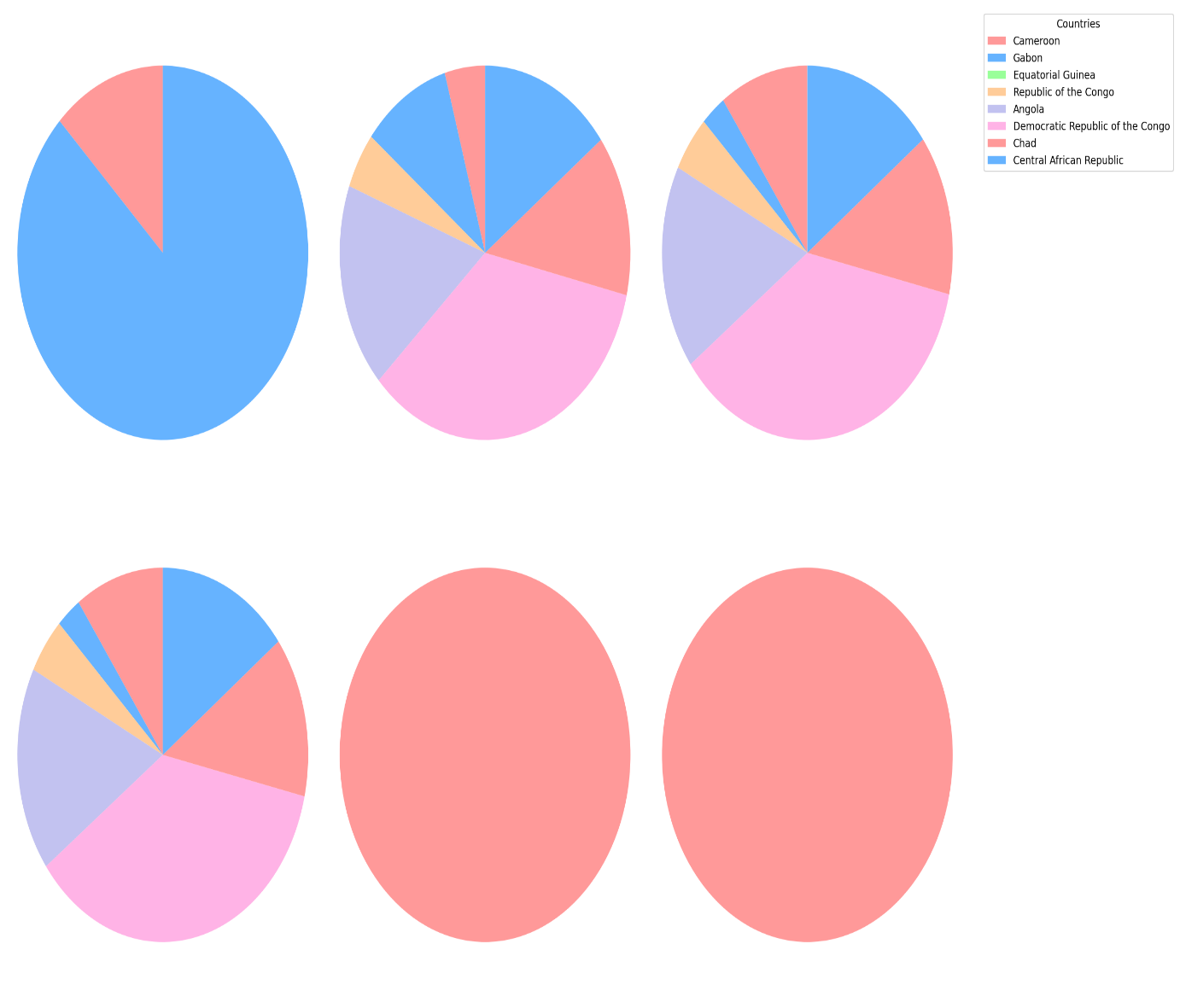


**Figure S7.** Pie charts displaying the distribution of highly suitable sites for countries in the Central Africa region for the solar pond development, showing the percentage of calculated area in hectares for six input combinations, arranged from left to right and top to bottom.


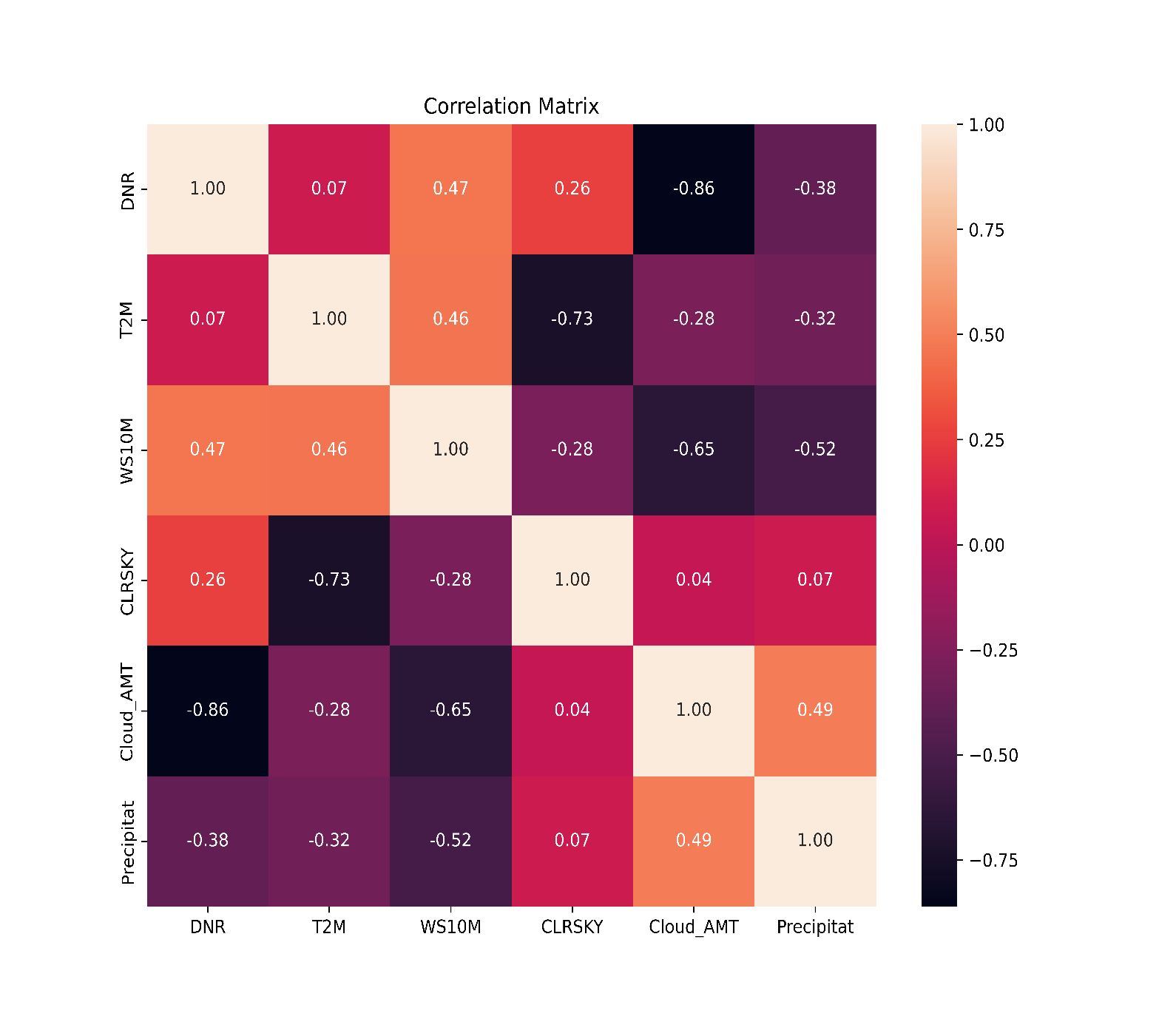


**Figure S8.** Correlation matrix for environmental remote sensing data for East Africa.


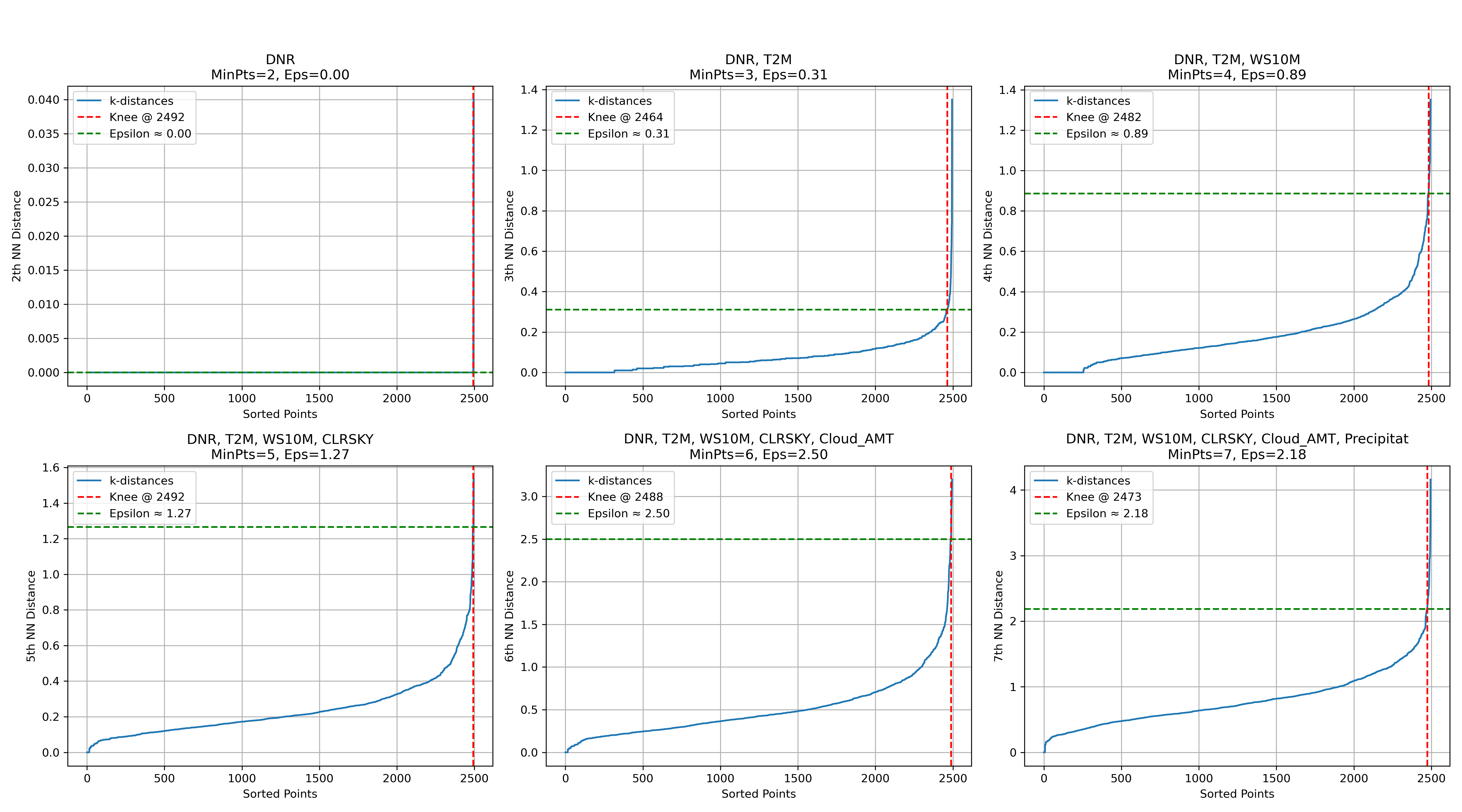


**Figure S9**. K-distance plots with minimum points and estimate epsilon values for six input combinations for East Africa.





**Figure S10.** Pie charts depicting the percentage distribution of calculated areas (in hectares) for East African countries identified as suitable for high-suitability solar pond development, based on six input combinations arranged from left to right and top to bottom.


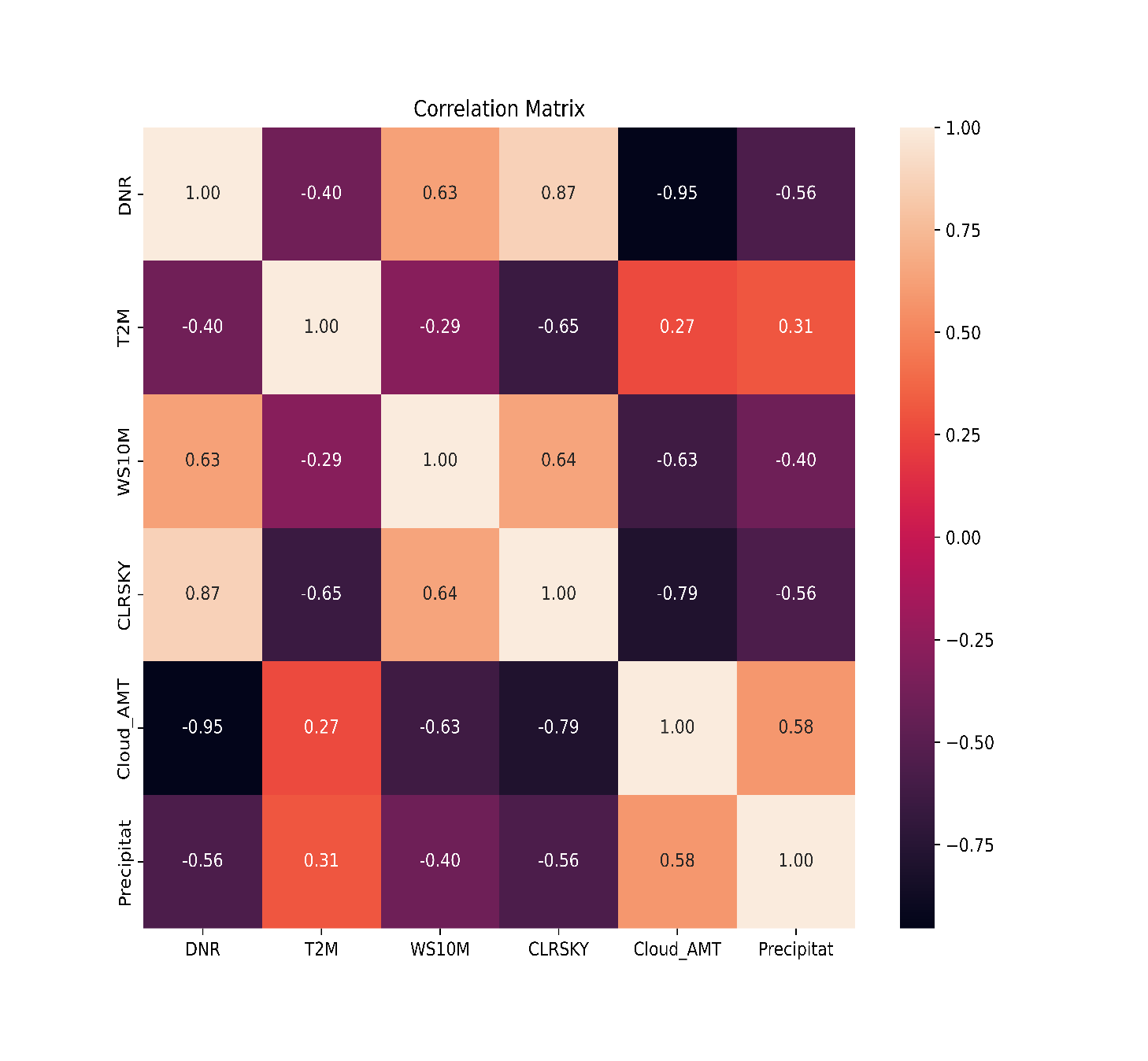


**Figure S11.** Correlation matrix for environmental remote sensing data for Southern Africa.


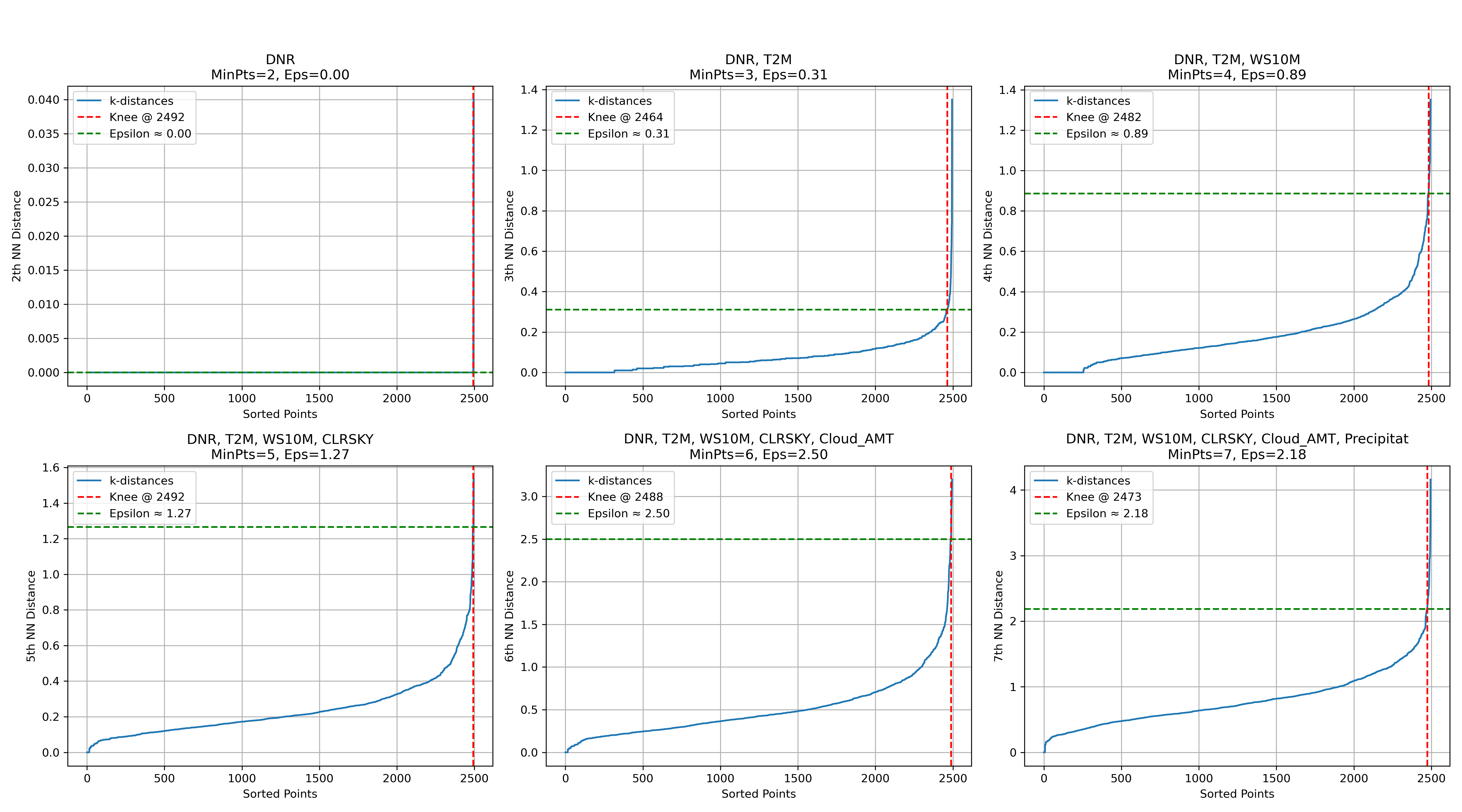


**Figure S12.** K-distance plots with minimum points and estimate epsilon values for six input combinations for Southern Africa.


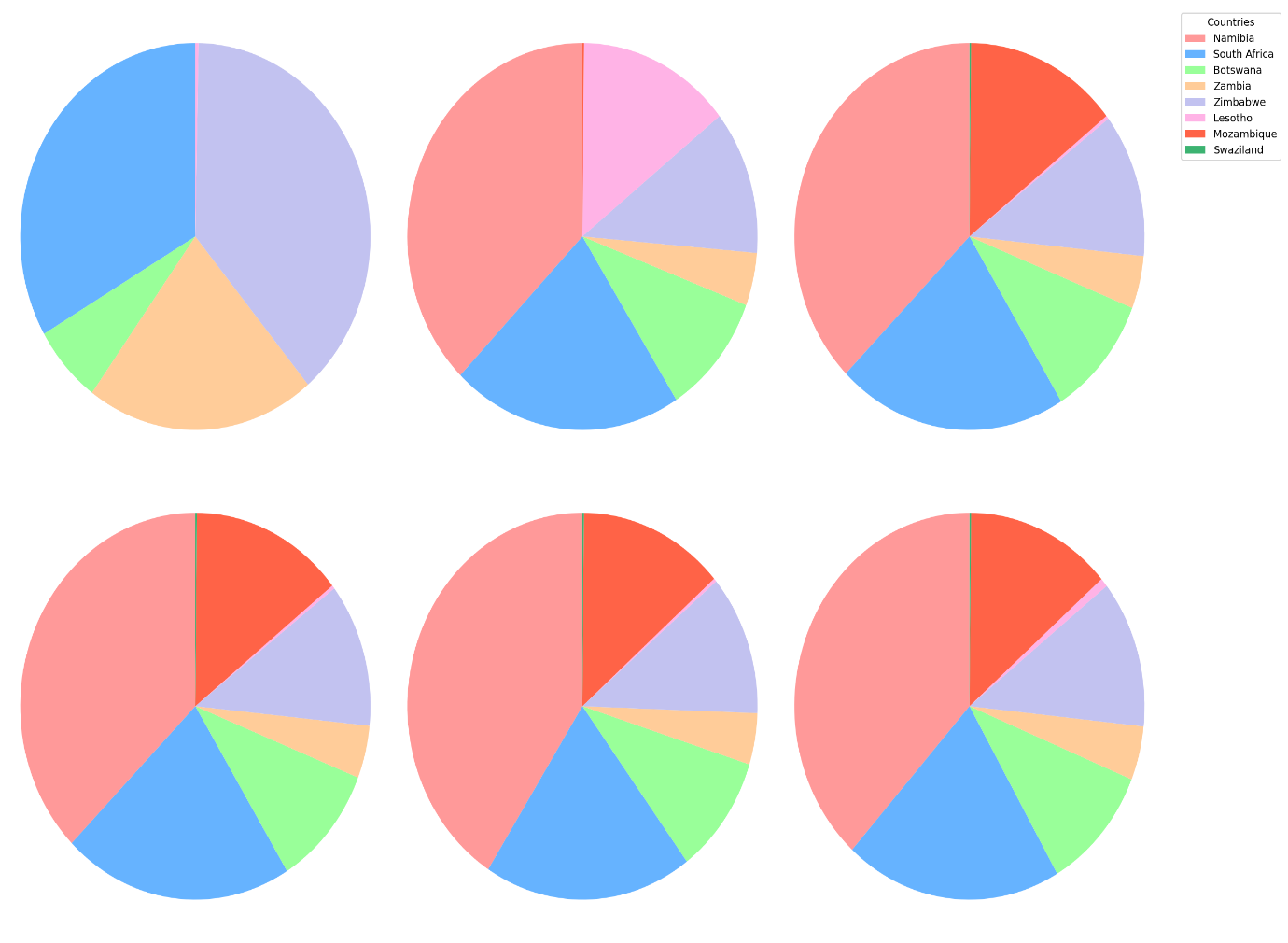


**Figure S13.** Pie charts showing the percentage distribution of highly suitable clusters for solar pond development site selection in Southern African countries, based on six input combinations arranged from left to right and top to bottom.


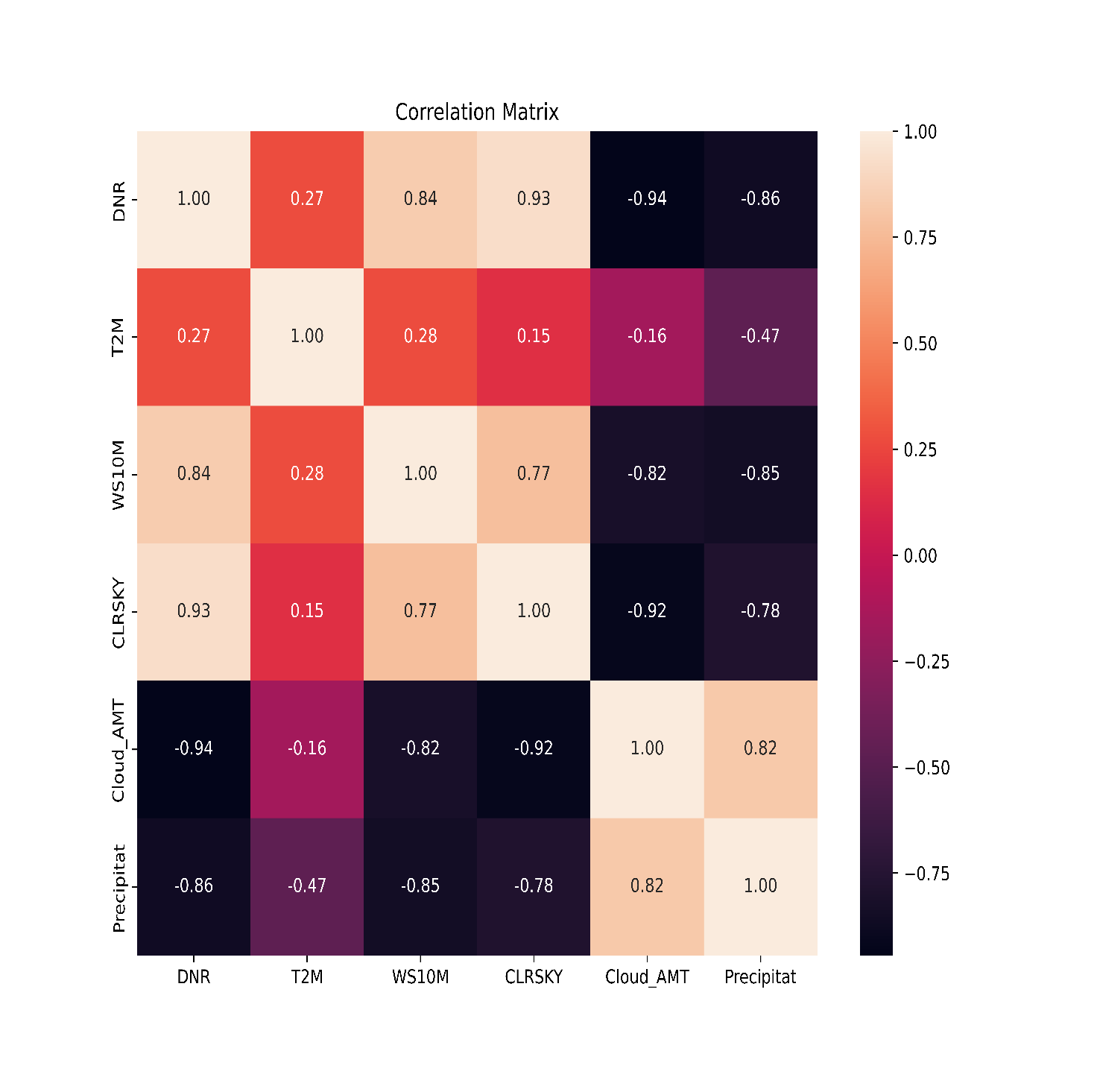


**Figure S14.** Correlation matrix for environmental remote sensing data for West Africa.


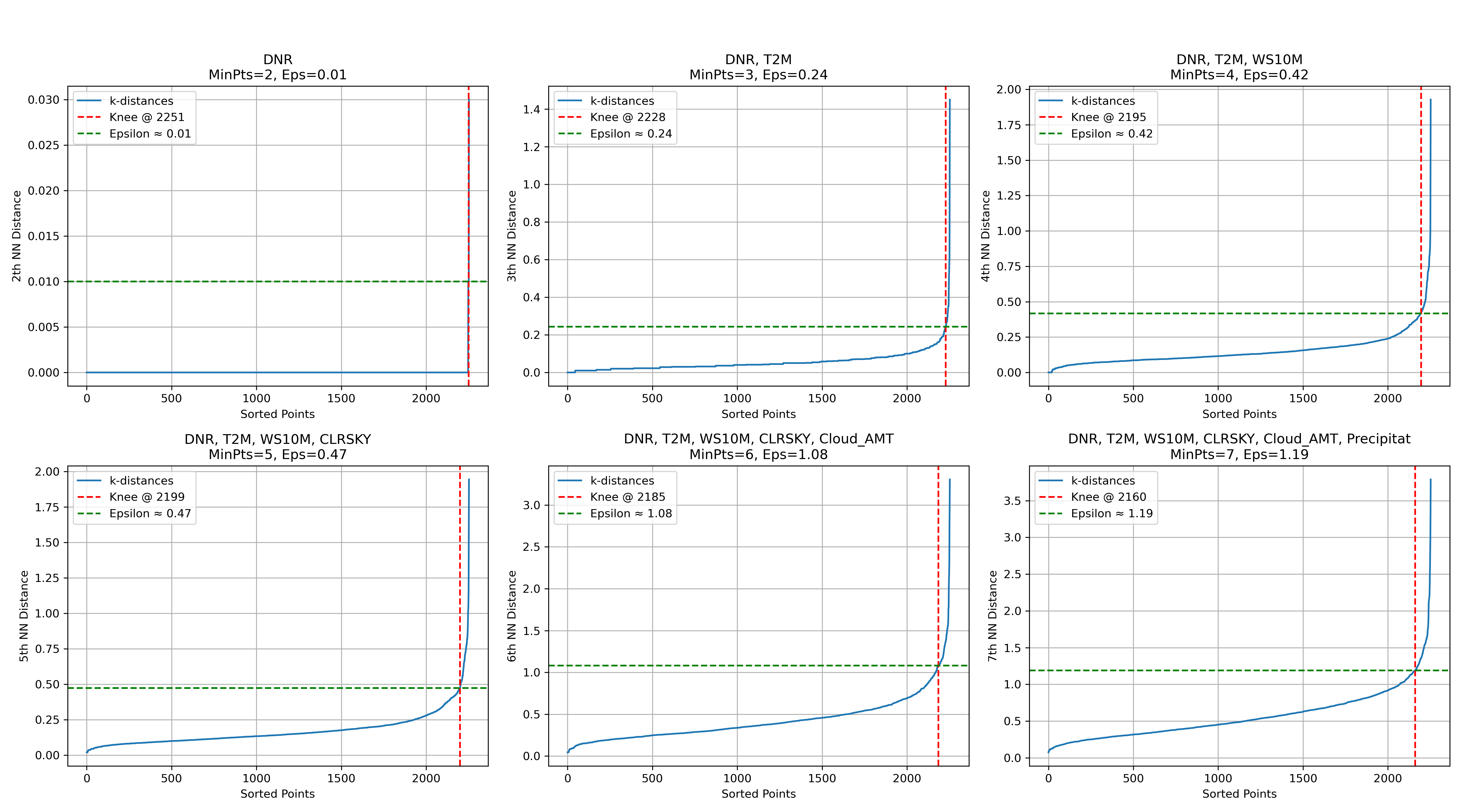


**Figure S15.**  K-distance plots with minimum points and estimate epsilon values for six input combinations for West Africa.


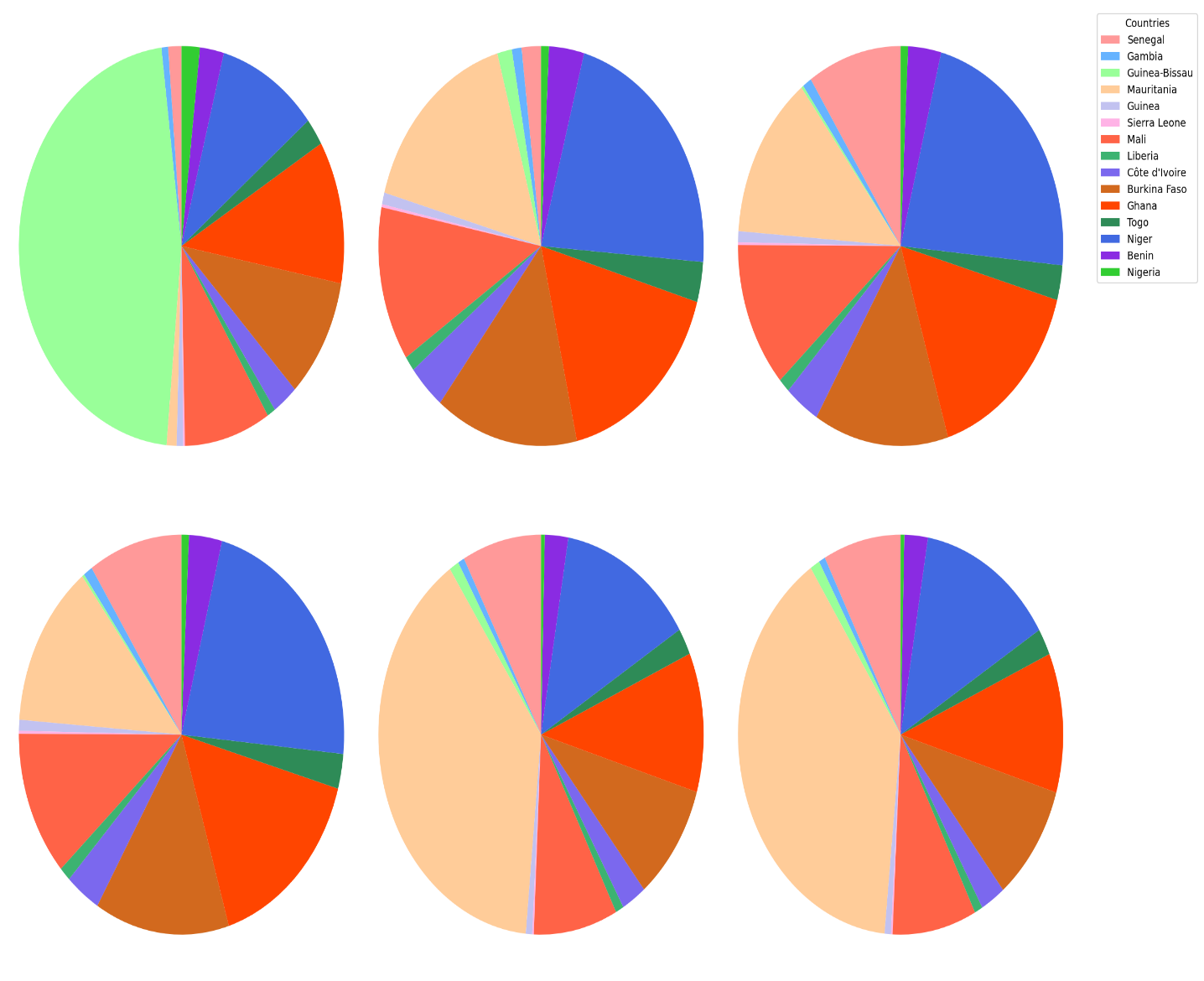


**Figure S16.** Pie charts showing the distribution of the high suitable area in hectares for the solar ponds development for countries in the West Africa region for different input combinations from the left to the right and from the top to the bottom.
